# Self-development inquiry (SDi) scale: a process-oriented measure of psychological integration in an organismic-transpersonal framework

**DOI:** 10.3389/fpsyg.2026.1834600

**Published:** 2026-07-29

**Authors:** Xavier Bronlet, Pier Luigi Lattuada

**Affiliations:** Integral Transpersonal Institute, Milan, Italy

**Keywords:** convergent validity, mediation, network psychometrics, organismic framework, psychological integration, structural equation modeling, systemic coherence

## Abstract

**Introduction:**

Psychological integration has been theorized across humanistic, neuroscientific, phenomenological, and complexity-based traditions as a multi-level process of experiential coordination, yet empirical operationalizations of integration as a structured organizational architecture remain limited. The present study introduces and validates the Self-Development Inquiry (SDi), a multidimensional instrument grounded in an organismic–transpersonal framework that models psychological functioning across structural resources (Q2), integration processes (Q1), and dysregulation-related signals (Q3).

**Methods:**

A community sample (*N* = 441) was analyzed under ordinal-appropriate estimation (WLSMV with polychoric correlations) using a 50/50 split-sample design (*N* = 220 exploratory; *N* = 221 confirmatory). Analyses comprised exploratory and confirmatory factor analysis, structural equation modeling with bootstrapped mediation, complementary network analysis of the dysregulation indicators, and convergent validity analysis against established benchmarks in a subsample (*N* = 76).

**Results:**

EFA and CFA supported a differentiated first-order structure with good approximate fit (CFI = 0.943, TLI = 0.933, RMSEA = 0.065, SRMR = 0.053), satisfactory composite reliability, and preserved discriminant validity. SEM identified a partially mediated hierarchical structure consistent with the theoretical architecture: physical–cognitive organization was strongly associated with external coherence (β = 0.82), which was in turn the dominant proximal correlate of both physical (β = −0.65) and mental disharmonies (β = −0.68). Bootstrapped mediation indicated robust indirect effects from structural resources to dysregulation via integration pathways, strongest through enacted congruence, while energetic organization retained additional direct regulatory effects. The model explained substantial variance in dysregulation outcomes (*R*^2^ = 0.71–0.72), with acceptable multicollinearity (all VIFs < 3) and stable estimates across split-sample validation. Network analysis of disharmonies revealed a densely interconnected, stable structure (CS-coefficient: strength = 0.52, expected influence = 0.67) with identifiable bridge nodes linking somatic and cognitive–existential indicators, consistent with conditional interdependence rather than reflective outputs of a single latent factor. Convergent validity analyses (*N* = 76) showed the four SDi LV2 composites correlated substantially and in predicted directions with the WHO-5 Wellbeing Index, the DASS-21, and the DERS, with all 24 primary correlations significant after Benjamini–Hochberg correction (|r| range = 0.39–0.82); the strongest associations fell on the most theoretically proximal benchmarks (wellbeing, depression), and facet-level analyses showed selective convergence (notably DERS Clarity, five facets at |*r*| ≥ 0.70) alongside near-zero correlations for theoretically distal pairs. Hierarchical regressions showed that integration and disharmonies blocks contributed robustly to predicting external scales, with the emotional resources facet adding substantial incremental variance to emotion-regulation prediction (Δ*R*^2^ = 0.078, *p* < 0.001).

**Discussion:**

Collectively, findings are consistent with conceptualizing psychological integration as a hierarchically organized system in which embodied structural capacities are associated with integrative coherence, which is in turn associated with reduced dysregulation. Because the design is cross-sectional, these associations do not permit inference about temporal direction. The convergent evidence supports the SDi as a theoretically coherent operationalization of integration that is empirically distinguishable from generalized affective distress while remaining substantively related to established wellbeing and emotion-regulation benchmarks. By integrating latent structural modeling with network psychometrics and convergent validity analysis, the SDi advances the empirical investigation of integration as a measurable organizational construct rather than a unidimensional wellbeing indicator.

## 1 Introduction

Psychological health is increasingly described not as the absence of symptoms, but as the presence of integration (Cloninger, 2006). Across psychotherapy, neuroscience, phenomenology, and transpersonal psychology, a convergent idea has emerged: wellbeing reflects the coordinated organization of differentiated experiential domains (Galvin and Todres, 2011). Affect, cognition, embodiment, relationality, and meaning must function in coherent alignment rather than in fragmentation or dissociation (Beckes et al., 2015).

Yet despite this growing theoretical convergence, integration remains conceptually rich but psychometrically underdeveloped (Carleton, 2012).

Humanistic psychology first articulated this problem in structural terms. Rogers’ concept of congruence framed distress as a mismatch between organismic experience and self-representation, while health was defined as alignment between Humanistic psychology provided one of the earliest systematic articulations of this principle. Rogers’ notion of congruence conceptualized distress as a mismatch between organismic experience and self-concept, while psychological health was defined as alignment between felt experience, awareness, and action (Rogers, 1961). Later organismic and developmental theories extended this view, proposing that psychological growth involves progressive coordination of increasingly differentiated aspects of the self (Wapner and Demick, 2013). In transpersonal traditions, integration is positioned as a developmental achievement that transcends ego-bound identity without dissolving structural coherence (Hartelius et al., 2013).

Parallel developments in neuroscience have reinforced this direction. Damasio’s somatic marker hypothesis demonstrated that cognition depends upon embodied affective integration (Damasio, 1994), with subsequent embodied-cognition research extending this view to demonstrate the role of bodily simulation in social and affective processing (Winkielman et al., 2008). Siegel (2001) proposed that mental health itself can be defined as integration: the linkage of differentiated neural and relational systems. Polyvagal-informed frameworks further situate psychological regulation within coordinated autonomic states that support relational safety and adaptive flexibility (Porges, 2025).

Phenomenological psychiatry adds another layer: distress is not simply a collection of symptoms but a reorganization of lived structure: alterations in temporality, embodiment, and relational orientation (Fuchs, 2020). Complexity theory similarly describes adaptive functioning as a higher-order attractor state emerging from dynamic coordination among interacting subsystems (Carmichael and Hadžikadić, 2019).

Across these domains, a shared conclusion becomes visible: psychological integration is a multi-level, self-organizing process characterized by differentiation without fragmentation and coherence without rigidity (diSessa et al., 2004).

Despite this theoretical richness, measurement practices have lagged. Most available instruments assess either:

- Subjective wellbeing (e.g., life satisfaction, positive affect),
- Symptom burden,
- Or spiritual/mystical experiences.

Few instruments directly operationalize integration as systemic coherence across structural, functional, and regulatory levels. The absence of such measures limits empirical testing of organismic and transpersonal theories and constrains process-oriented psychotherapy research.

The present study addresses this gap through the development of the Self-Development inquiry (SDi). The SDi operationalizes psychological integration within a hierarchical organismic–transpersonal architecture composed of three interrelated levels:

- Q2—Structural Organismic Resources, reflecting organized capacities across physical, energetic, emotional, mental, and spiritual domains.
- Q1—Experiential Integration, capturing functional coherence across cognitive, somatic, relational, and internal processes.
- Q3—Disharmonies, representing phenomenological signals of systemic dysregulation.

In this framework, integration is conceptualized not as elevated mood or spiritual intensity, but as an organizational property of experience. Structural resources provide capacity; integration processes translate capacity into coherence; disharmonies signal breakdowns in regulatory alignment. The resulting model permits empirical examination of a hierarchical mediation logic consistent with organismic self-regulation, embodied cognition, and complexity-based accounts of adaptive functioning.

Accordingly, the present study investigates whether the SDi demonstrates valid and reliable measurement of psychological integration as a multidimensional systemic construct. Specifically, the study tested whether:

a) Organismic structural resources are positively associated with integration domains.
b) Integration domains are negatively associated with disharmonies.
c) Integration statistically accounts for (i.e., partially mediates) the association between organismic resources and disharmonies.
d) These relations are coherent across domain-specific pathways within the proposed architecture.

By integrating exploratory refinement, confirmatory modeling, and hierarchical mediation analysis, the study seeks to move integration from a conceptual ideal to an empirically testable architecture (Ciptono et al., 2010). In doing so, it contributes both a psychometric instrument and a structural model capable of advancing organismic–transpersonal research.

## 2 Literature review

### 2.1 Psychological integration as multi-level coherence

#### 2.1.1 From congruence to embodied integration

The concept of psychological integration has evolved across multiple theoretical traditions but converges on a shared core premise: wellbeing is not merely the absence of symptoms but the dynamic coordination of affective, cognitive, somatic, and behavioral systems (Cloninger, 2006). Integrative models describe psychological health as coherence across differentiated experiential domains, rather than reduction to a single functional dimension (Arvidsdotter et al., 2015).

Humanistic psychology provided one of the earliest systematic articulations of this principle. Rogers’ notion of congruence conceptualized distress as a mismatch between organismic experience and self-concept, while psychological health was defined as alignment between felt experience, awareness, and action (Rogers, 1961). Within this perspective, psychological functioning can be conceptualized as the dynamic coordination of affective, cognitive, and behavioral systems, where wellbeing reflects coherent integration across these domains rather than the absence of symptoms; when these domains become misaligned, individuals may experience internal conflict, reactive behavior, and diminished regulatory capacity (Rogers, 1961). Gendlin (1991) extended this position through the concept of the felt sense, emphasizing that cognitive meaning-making must remain grounded in embodied experiencing to preserve integration.

These perspectives reposition the self not as a static entity but as an emergent regulatory process. Integration becomes an ongoing, organismic coordination rather than a fixed structural property (Marks-Tarlow, 1999).

#### 2.1.2 Neuroscientific foundations of integration

Neuroscientific research further substantiates integration as a core mechanism of psychological functioning (Solms and Turnbull, 2018). Damasio’s somatic marker hypothesis proposed that affective and bodily states guide reasoning and decision-making, with dissociation between emotional and cognitive systems compromising adaptive functioning (Damasio, 1994). Embodied cognition models extend this view, emphasizing that adaptive functioning depends on the integration of bodily signals with cognitive processes; emotional and somatic feedback contribute to decision-making and behavioral regulation, with disruptions in this integration potentially impairing coherent action and psychological adjustment (Winkielman et al., 2008).

Siegel’s definition of mental health as “integration” frames wellbeing as the linkage of differentiated neural and relational systems, with chaos and rigidity representing complementary forms of dysregulation (Siegel, 2001). Similarly, polyvagal theory situates psychological regulation within neurophysiological states of safety and co-regulation, suggesting that coherent action presupposes autonomic integration (Porges, 2025).

Across these models, integration is characterized by:

- Differentiation without fragmentation
- Flexibility without instability
- Regulation without suppression

#### 2.1.3 Phenomenological psychiatry and structural organization

Phenomenological psychiatry provides a complementary lens by conceptualizing both wellbeing and distress as structured modes of experiencing rather than as linear opposites (Fuchs, 2020). Jaspers, Minkowski, and Binswanger emphasized disturbances in lived time, embodiment, and relationality as core features of psychopathology (Stanghellini et al., 2019).

Fuchs’ embodied phenomenology further develops this perspective by describing distress as a global reorganization of experiential structure rather than a localized dysfunction. This view aligns with the notion of phenomenological isomorphism: wellbeing and distress share structural dimensions but differ in orientation toward integration versus fragmentation (Fuchs, 2020).

#### 2.1.4 Salutogenic and multidimensional wellbeing models

The salutogenic framework introduced by Antonovsky reframed health as a function of *sense of coherence*, defined by comprehensibility, manageability, and meaningfulness (Antonovsky, 1987). Similarly, multidimensional wellbeing models conceptualize flourishing as active organization across autonomy, relational depth, and purpose (Ryff, 1989; Keyes, 2002). Eudaimonic models of wellbeing conceptualize flourishing as a multidimensional configuration including autonomy, relational depth, purpose, and personal growth, emphasizing that psychological health reflects coordinated functioning across multiple domains of human experience (Ryff, 1989). Recent reviews confirm this multidimensional structure across clinical and non-clinical populations (Brandel et al., 2017).

Research on the mental health continuum further suggests that flourishing and psychological distress represent distinct organizational states rather than opposite ends of a single dimension. Individuals may therefore experience varying configurations of wellbeing and distress across psychological domains (Keyes, 2002).

Crucially, these approaches conceptualize wellbeing as structured coherence rather than emotional positivity alone (Waters and Fivush, 2015). This distinction is particularly relevant for integrative models that treat psychological development as progressive alignment among experiential domains.

#### 2.1.5 Complexity and self-organization

Complexity theory provides a meta-theoretical frame for integration. Prigogine’s work on order emerging from instability (Prigogine and Stengers, 1984) and Maturana and Varela’s concept of autopoiesis (Maturana and Varela, 1980) suggest that living systems self-organize through recursive interaction with their environments

Within this perspective, integration represents a higher-order attractor state of experiential systems. Distress, conversely, reflects constricted or rigidified attractor dynamics (Firaux et al., 2025).

Complexity frameworks conceptualize psychological functioning as a self-organizing system in which patterns of experience emerge from interactions among cognitive, affective, and relational processes. From this perspective, both wellbeing and distress can be understood as distinct attractor states within evolving experiential systems (Prigogine and Stengers, 1984).

Taken together, psychological, neuroscientific, phenomenological, and complexity-based models converge on a shared conceptualization: psychological integration is a multi-level, self-organizing process characterized by embodied coherence across affect, cognition, relationality, and action (Fuchs, 2020; Porges, 2025; Siegel, 2001).

### 2.2 Subjective experience, quality of experience, and measurement

#### 2.2.1 Conceptual ambiguities in subjective wellbeing

The empirical assessment of integration requires engagement with the literature on subjective wellbeing (SWB). However, the conceptual foundations of SWB remain contested. Angner, 2010 highlights that scholars diverge on whether subjective wellbeing constitutes wellbeing simpliciter or represents only a component of it (2010).

This distinction is critical. Some traditions equate wellbeing with reported happiness or life satisfaction. Others treat subjective reports as partial indicators of broader structural organization (Andrews and Robinson, 1991).

Furthermore, subjective wellbeing itself includes heterogeneous components (Tatarova et al., 2021):

- Cognitive evaluation (life satisfaction)
- Hedonic tone (pleasure–pain balance)
- Affective frequency/intensity

These components may not fully capture embodied or relational integration.

#### 2.2.2 Quality of experience (QoE) and multi-contextual processing

Research on Quality of Experience (QoE) expands the evaluative framework by analyzing how perception, contextual factors, and cognitive appraisal interact in the formation of experienced quality (Raake and Egger, 2014).

QoE research distinguishes between:

- Immediate perceptual experiencing
- Higher-order evaluative judgment
- Contextual embedding (situational, socio-cultural, interactional)

This distinction parallels integration theory: subjective judgment emerges from layered experiential processing rather than from isolated affective states. QoE frameworks emphasize that quality judgments are constructed within dynamic contexts, reinforcing the idea that integration must be assessed as a structured, situated process (Raake and Egger, 2014).

#### 2.2.3 Embodied and organismic perspectives

Embodied psychotherapy traditions further contribute to this discourse by framing self-regulation as organismic coordination between somatic, affective, and cognitive processes (Krueger, 2013). Historical organismic models (e.g., Goldstein; Reichian traditions) conceptualize health as functional integration rather than symptom suppression (Sletvold, 2014). Embodied cognition perspectives further emphasize that mind and experience arise through continuous interaction between organism and environment. Psychological processes therefore cannot be fully understood independently of bodily engagement and relational context (Varela et al., 1991).

Embodied perspectives integrate affect regulation, somatic awareness, and relational attunement into unified models of psychological coherence (Rappoport, 2015), with subsequent work emphasizing the role of interoceptive accuracy and emotional differentiation in adaptive functioning (Khalsa et al., 2018; Barrett, 2017).

#### 2.2.4 From symptom reduction to structural organization

Across these literatures, a conceptual shift is evident:

- From pathology to organization
- From symptom counts to experiential structure
- From isolated traits to dynamic coherence

An integrative assessment model must therefore capture:

1. Structural organization across domains
2. Regulation capacity
3. Narrative coherence
4. Embodied congruence
5. Relational integration

This theoretical background justifies multidimensional measurement approaches capable of distinguishing fragmentation from integration without reducing the construct to mood or satisfaction alone.

## 3 Positioning of the present study

The present study builds upon this transdisciplinary convergence. It conceptualizes psychological integration as a multi-level organizational architecture composed of structurally distinct yet systematically related domains (e.g., self-regulation, interpersonal coherence, embodied alignment).

By integrating structural modeling with domain-level mediation analyses, the study empirically tests whether integration operates as a multi-level rather than as a unidimensional organization.

## 4 The organismic–transpersonal architecture of psychological integration

Building upon the transdisciplinary literature reviewed above, the present framework conceptualizes psychological integration as a hierarchical, organismic process operating across structural, functional, and signals levels. Rather than treating resources and distress as categorical opposites, the SDi model assumes that both emerge from the dynamic organization of experiential systems. Integration is therefore understood not as the absence of symptoms, but as systemic coherence within a multi-layered organismic architecture.

### 4.1 Structural level: organismic resources (Q2)

At the foundational level, the model posits the existence of organismic structural resources. These resources correspond to relatively stable domains of self-organization (physical, energetic, emotional, mental, and spiritual) which together constitute the organismic resources.

In this framework, the organismic resources is a self-organizing regulatory process through which embodied feeling, cognition, emotion, energy and sense of self progressively align. Structural resources represent the available capacity of the system. When sufficiently developed, they provide the stability necessary for adaptive self-regulation. When underdeveloped or fragmented, they constrain the system’s capacity for coherent integration.

Thus, Q2 does not measure subjective states but structural resources: the degree to which the individual possesses organized internal resources across organismic domains.

### 4.2 Functional level: experiential integration (Q1)

If structural resources represent the organismic substrate, experiential integration represents its functional expression. Integration is conceptualized as the dynamic coherence across cognitive, somatic, relational, and internal domains of experience.

At this level, integration manifests as:

- ability to sustain experiences,
- embodied awareness,
- relational attunement,
- narrative coherence,
- adaptive regulation.

Integration functions as a mediating regulatory process translating structural capacity into lived coherence. It reflects the system’s ability to coordinate differentiated components without collapsing into rigidity or fragmentation. In complexity terms, integration corresponds to a flexible attractor state characterized by dynamic stability.

In that sense, integration is an organizational property of experience.

### 4.3 Regulatory-signal level: disharmonies (Q3)

The third level concerns disharmonies. Within this model, disharmonies are not conceptualized as pathological defects but as regulatory signals indicating systemic overload, fragmentation, or compensatory imbalance.

Disharmonies represent the phenomenological markers of incoherence. They may manifest somatically, emotionally, cognitively, or existentially. However, they are interpreted as expressions of system-level dysregulation rather than isolated symptoms.

This perspective aligns with phenomenological psychiatry and systemic models in which distress reflects reorganization of experiential structures rather than mere malfunction (Fuchs, 2020). Disharmonies thus serve as indicators of misalignment within the organismic hierarchy.

### 4.4 Hierarchical mediation logic

The theoretical architecture implies a directional relationship among these three levels:

1. Structural resources (Q2) provide organismic capacity.
2. Integration processes (Q1) translate capacity into coherence.
3. Disharmonies (Q3) emerge when integration fails or becomes overloaded.

Accordingly, integration is hypothesized to statistically account for the association between organismic resources and disharmonies within a hierarchical structural model. The framework conceptualizes structural robustness as a necessary but not sufficient condition for adaptive functioning: structural resources are theorized to support the system’s capacity for integration, while integration itself is theorized to be associated with reduced disharmony. The cross-sectional design of the present study cannot establish the temporal or causal direction of these relations; the architecture is therefore tested as a structural hypothesis about cross-sectional associations rather than as a causal claim about temporal dynamics.

This hierarchical logic is consistent with organismic resources-regulation theory, embodied cognition models, and complexity-based accounts of adaptive systems. It also bridges humanistic congruence theory (Thorne, 2003) and transpersonal integration models by situating transformation within a measurable structural–process architecture (Giesinger, 2013).

### 4.5 Implications for measurement

The SDi operationalizes this architecture through three distinct yet interrelated measurement blocks:

- Q2 captures organismic structural resources.
- Q1 captures integration functional coherence.
- Q3 captures dysregulation signals.

Unlike wellbeing scales (which assess positive outcomes), symptom scales (which assess distress), or spiritual inventories (which assess experiences), the SDi measures systemic organization across levels. This makes it particularly suited for testing process-oriented and mediation-based hypotheses within an organismic–transpersonal framework.

Given ongoing debates regarding whether dysregulation phenomena are best conceptualized as reflective indicators or as mutually interacting regulatory signals, complementary modeling approaches may be warranted when examining disharmonies.

In summary, the SDi is not merely intended as a scale, but as an operationalization of a hierarchical organismic model in which structural resources, integrative processes, and dysregulation signals form a coherent dynamic system. This architecture provides the theoretical foundation for the structural equation modeling tested in the subsequent sections.

### 4.6 Hypotheses

Building on the organismic–transpersonal architecture outlined above, the present study advances five hypothesis blocks corresponding to the structural relations among Q2 (organismic resources), Q1 (integration processes), and Q3 (disharmonies). Each hypothesis specifies an expected direction grounded in the theoretical framework, allowing the structural model to be evaluated against pre-specified directional predictions rather than as a purely exploratory enterprise.

#### 4.6.1 Block 1—resources predict integration (Q2 → Q1)

*H1a*: Physical–cognitive organization (SE_FIS) is positively associated with mental integration (ISO_MEN).

*H1b*: Emotional organization (SE_EMO) is positively associated with mental integration (ISO_MEN).

*H1c*: Physical–cognitive organization (SE_FIS) is positively associated with external coherence (ISO_CEX).

*H1d*: Spiritual organization (SE_SPI) is positively associated with internal coherence (ISO_CIN).

Rationale: Within the SDi architecture, structural resources scaffold integration through domain-specific channels. Embodied stability and cognitive clarity (SE_FIS) underpin the reflective processing required for mental integration and the behavioral enactment that constitutes external coherence. Emotional differentiation (SE_EMO) supports reflective regulation, contributing to mental integration. Spiritual orientation (SE_SPI) provides the existential grounding underlying internal coherence.

#### 4.6.2 Block 2—integration predicts dysregulation (Q1 → Q3)

*H2a*: External coherence (ISO_CEX) is negatively associated with both physical and mental disharmonies (DIS_FIS, DIS_MEN).

*H2b*: Mental integration (ISO_MEN) is negatively associated with mental disharmony (DIS_MEN).

Rationale: Integration processes act as proximal regulators of dysregulation. External coherence (the alignment between thought, feeling, and action) functions as the dominant integrative buffer against both somatic and cognitive–existential disharmony. Mental integration further constrains the propagation of cognitive–existential dysregulation through reflective stabilization.

#### 4.6.3 Block 3—indirect effects (Q2 → Q1 → Q3)

*H3*: The associations between organismic resources (Q2) and disharmonies (Q3) are partially mediated by integration processes (Q1), with structural resources operating both directly and indirectly via the integrative pathways specified in H1 and H2.

Rationale: The theoretical architecture specifies that integration processes translate structural capacity into adaptive functioning. Effects of resources on dysregulation should therefore be partly transmitted through integration, while leaving room for direct embodied or energetic effects that bypass reflective integration.

#### 4.6.4 Block 4—direct resource–dysregulation associations

*H4a*: Energetic organization (SE_ENE) is directly negatively associated with physical disharmony (DIS_FIS).

*H4b*: Energetic organization (SE_ENE) is directly negatively associated with mental disharmony (DIS_MEN).

*H4c* (exploratory): The direct association between emotional organization (SE_EMO) and physical disharmony (DIS_FIS) is examined without a directional prediction. Both protective (negative) and inconsistent-mediation (positive) patterns are theoretically defensible, given that emotional differentiation may simultaneously support reflective regulation (negative indirect via ISO_MEN) and heighten interoceptive sensitivity to somatic strain (positive direct).

Rationale: Vitality-related processes (SE_ENE) are theorized to influence dysregulation through both integrative and direct embodied channels (e.g., autonomic regulation, physiological energy) that are not fully captured by reflective integration. Emotional organization is treated exploratorily because the literature does not converge on a single expected sign for the direct path: heightened emotional awareness may either buffer or amplify somatic dysregulation depending on integrative capacity.

#### 4.6.5 Block 5—residual covariance among outcomes

*H5*: Physical and mental disharmonies (DIS_FIS, DIS_MEN) share residual covariance, reflecting unmodeled common variance in dysregulation that is not accounted for by upstream structural and integrative predictors.

Rationale: Within an organismic framework, somatic and cognitive–existential disharmonies are expected to co-occur and reinforce one another beyond what structural and integrative predictors alone explain. This residual association is modeled explicitly as a covariance rather than as a structural effect, consistent with the assumption that dysregulation signals form a distributed and partially self-reinforcing system (further examined via network analysis).

Together, these five hypothesis blocks specify directional predictions for each estimated path in the structural model. Hypotheses H1a–H1d, H2a–H2b, H3, H4a–H4b, and H5 are pre-specified directional tests; H4c is explicitly identified as exploratory. The structural model and mediation analyses reported in the Results section evaluate each of these predictions.

## 5 Methodology

### 5.1 Design and analytic strategy

This study employed a cross-sectional psychometric validation design to evaluate the factorial structure, reliability, construct validity, and structural relations of the SDi. Analyses integrated latent variable modeling and network psychometrics to test distinct structural assumptions across domains.

Organismic resources (Q2) and integration processes (Q1) were modelled as reflective latent variables within a structural equation modling (SEM) framework. Disharmonies (Q3) were examined both as latent variables and, complementarily, using network modeling to assess potential conditional interdependence among dysregulation indicators.

Given the ordinal nature of the 5-point Likert items, all confirmatory factor analyses and structural equation models were estimated with the robust mean- and variance-adjusted weighted least squares estimator (WLSMV) on a polychoric correlation matrix, in line with current methodological recommendations for ordinal indicators (Li, 2016; Wu and Estabrook, 2016). Maximum likelihood with robust standard errors (MLR) was retained as a supplementary estimator for analyses requiring likelihood-based output (e.g., bootstrap-based confidence intervals for indirect effects), with results compared across estimators to assess robustness. All analyses were conducted in R.

### 5.2 Item development

The SDi was developed natively in Italian by the authors, drawing on the organismic–transpersonal framework articulated in the Theoretical Architecture section. Items were not adapted or translated from existing instruments; they were authored to operationalize the three-block architecture (Q1 Experiential Integration, Q2 Organismic Resources, Q3 Disharmonies) at the construct level appropriate to each block. The drafting process proceeded in three phases.

First, an initial item pool was generated to cover the conceptual scope of each Q-block as defined in the theoretical framework, with explicit attention to balance across the targeted sub-domains (e.g., for Q2: physical, energetic, emotional, mental, and spiritual; for Q1: cognitive, somatic, relational, and internal coherence; for Q3: somatic and cognitive–existential signals). Items were written to capture process-level expressions of integration rather than dispositional states or symptomatic outcomes, in keeping with the organismic conceptualization advanced in the framework.

Second, the initial item pool was submitted to expert review by six colleagues at the Integral Transpersonal Institute with backgrounds in transpersonal psychology, psychotherapy, and psychometric assessment. Reviewers evaluated each item for theoretical fidelity to the organismic–transpersonal framework, semantic clarity, and conceptual fit to its intended Q-block. Items judged to lack theoretical fidelity, to be ambiguous in wording, or to overlap excessively with items in other blocks were revised iteratively until consensus was reached among the review panel.

Third, the resulting questionnaire was pilot-tested with a sample of 25 Italian-speaking adults prior to deployment in the formal study. The pilot phase verified the comprehensibility of items, the time required for completion, and the absence of systematic patterns of non-response. No substantive item revisions were required following the pilot; minor adjustments to instructions and presentation were incorporated into the final questionnaire administered in the present study.

The final questionnaire administered to participants comprised 45 items distributed across the three Q-blocks (Q1: 15 items; Q2: 15 items; Q3: 15 items). The Q-block ordering reflects the administered sequence of the questionnaire (integration processes presented first, organismic resources second, disharmonies third); this administration order is distinct from the theoretical sequence implied by the structural model (resources → integration → disharmonies), and the latter is reflected in the directional structural specification rather than in the Q-block numbering. Items were rated on a 5-point Likert scale (1 = *Mai*/Never; 5 = *Sempre*/Always). Three items in Q1 (sdi_13, sdi_14, sdi_15) were worded such that higher endorsement corresponds to lower integration; these items were reverse-coded prior to all analyses so that higher scores on every item correspond to greater integration, greater organismic resources, or (for Q3 only) greater disharmony. The complete item list, including original Italian wording and English translations, is reported in Tables 3–5 within the Results section.

### 5.3 Participants and procedure

The sample consisted of Italian speaking adults recruited via online voluntary participation. Participants completed the SDi anonymously using an online survey platform after providing informed consent. No missing data were observed across the SDi items. Response scale and item reversal are described in the Item Development subsection above.

### 5.4 Exploratory and confirmatory factor analyses

To guard against the inflation of model fit that can result from item refinement and structure validation on the same sample, the dataset (*N* = 441) was randomly split into two halves prior to analysis. The exploratory half (*N* = 220) was used to conduct exploratory factor analyses and item refinement; the confirmatory half (*N* = 221) was used to evaluate the resulting structure via confirmatory factor analysis. The split was performed with a fixed random seed for full reproducibility. Confirmatory analyses were additionally re-estimated on the full sample to retain continuity with the original derivation and to assess whether parameter estimates were stable across both subsamples (Cabrera-Nguyen, 2010).

Exploratory factor analyses (principal axis factoring, oblimin rotation) were conducted on the exploratory half separately for Q1, Q2, and Q3, using a polychoric correlation matrix appropriate for ordinal indicators. Factor retention was based on parallel analysis on polychoric correlations, scree inspection, and theoretical interpretability. Items were retained if primary loadings were ≥ 0.40 and cross-loadings were < 0.30.

The refined structure was evaluated on the confirmatory half (*N* = 221) using confirmatory factor analysis (CFA) with the WLSMV estimator on a polychoric correlation matrix. To assess parameter stability and to maintain continuity with the original derivation sample, the same models were re-estimated on the full sample (*N* = 441), with both sets of fit indices reported. Model fit was assessed using the scaled χ^2^, CFI, TLI, RMSEA (90% CI), and SRMR. Conventional thresholds for acceptable fit were CFI/TLI ≥ .90 (excellent ≥ 0.95), RMSEA ≤ 0.08 (excellent ≤ 0.06), and SRMR ≤ 0.08 (Hu and Bentler, 1999; Kline, 2023). Composite reliability (ω) and Average Variance Extracted (AVE) were computed from the standardized solution. Two latent factors retained from the EFA solution were defined by two indicators (ISO_CIN: sdi_12, sdi_13; ISO_PER: sdi_1, sdi_5); to identify the resulting two-indicator factors, equality constraints on factor loadings were imposed within each two-indicator factor (Bollen, 1989; Kline, 2023), an established practice that does not affect the interpretation of standardized solutions.

To evaluate the dimensional structure of the SDi against plausible alternatives, four competing measurement models were estimated and compared on the full sample using WLSMV: (a) a unidimensional model with all items loading on a single general factor; (b) the first-order correlated factor model corresponding to the hypothesized 10-factor structure; (c) a second-order model in which three higher-order factors (organismic resources, integration, dysregulation) account for the covariation among first-order factors; and (d) a bifactor model with a general factor and orthogonal specific factors. Models were compared using the scaled χ^2^ difference test for nested specifications (Satorra and Bentler, 2010) and on conventional fit indices.

### 5.5 Structural model

A structural model was specified in which organismic resources (Q2) were modeled as predictors of integration processes (Q1), which in turn were modeled as predictors of disharmonies (Q3). Selected direct paths from Q2 to Q3 were retained where theoretically justified and empirically supported. The directional terminology (*predict*) refers throughout to regression coefficients in the structural specification and does not imply a temporal-causal claim; the cross-sectional design does not support such inference.

The structural model was estimated with WLSMV on a polychoric correlation matrix. Indirect effects were tested using nonparametric bootstrap with 5,000 resamples and bias-corrected and accelerated (BCa) confidence intervals; because lavaan does not currently support se = “bootstrap” under WLSMV, the bootstrap step was performed using MLR for inferential purposes, while parameter estimates and Delta-method standard errors were obtained from the primary WLSMV fit. Nested mediation models were compared using scaled χ^2^ difference testing (Satorra and Bentler, 2010). Variance explained (R^2^) was reported for endogenous constructs. Multicollinearity was evaluated using variance inflation factors (VIF) (Gunzler et al., 2013).

The structural model was specified in two stages. The full structural model included all theoretically motivated paths corresponding to the hypothesis blocks specified above, and was used to test the hypothesis pattern as a whole. A reduced (trimmed) structural model was then estimated, retaining only paths meeting both of two criteria: a standardized coefficient magnitude | β| ≥ 0.10 and a statistical significance threshold of *p* < 0.05 in the full model. The two-criterion rule excluded paths that were either statistically detectable but practically negligible (| β| < 0.10) or large but unstable (*p* ≥ 0.05), and was applied to all directional structural paths but not to factor loadings, covariances among exogenous resources, or the residual covariance specified by H5. The trimmed model is reported as the primary structural specification; full-model coefficients are available from the corresponding author upon reasonable request.

### 5.6 Measurement invariance

Multi-group invariance across gender (women, men) was evaluated under both the primary ordinal estimator (WLSMV with delta parameterization on a polychoric correlation matrix) and a supplementary continuous estimator (MLR), in line with current recommendations for ordinal data (Wu and Estabrook, 2016). Three nested models were fitted: configural (no constraints), metric (equal loadings; under WLSMV, equal loadings and thresholds), and scalar (additionally equal intercepts). Invariance was evaluated using fit-index change criteria (ΔCFI ≤ 0.010, ΔRMSEA ≤ 0.015; Chen, 2007) for the primary WLSMV analysis, with the scaled χ^2^ difference test (Satorra and Bentler, 2010) reported for the supplementary MLR analysis. For the WLSMV analysis, items with empty response categories in one gender group were collapsed by combining the bottom two response categories uniformly across both groups, in line with standard practice for ordinal multi-group invariance (Wu and Estabrook, 2016; Liu et al., 2017). The participant who selected “other” for gender (*n* = 3) was excluded from invariance analyses due to insufficient cell size.

### 5.7 Network analysis of disharmonies

To examine whether disharmonies exhibit conditional interdependence beyond latent variable assumptions, a Gaussian Graphical Model (GGM) was estimated for Q3 indicators using EBICglasso regularization on a polychoric correlation matrix automatically detected for the ordinal items (qgraph::cor_auto). Edges represent regularized partial correlations. Network stability was assessed via nonparametric and case-dropping bootstrap procedures (1,000 resamples each), with the Correlation Stability (CS) coefficient computed for strength and expected influence centrality (Epskamp et al., 2018). Node strength and bridge strength (Jones et al., 2018) were examined descriptively.

Two methodological cautions inform the interpretation. First, networks estimated from cross-sectional data describe regularized conditional associations among items at a single time point and do not support causal or directional inference. Bridge centrality, in particular, identifies items that are conditionally associated with multiple communities of nodes, but does not imply that activation of a bridge node propagates causally between communities. Second, the EBICglasso estimator is sensitive to the choice of the regularization parameter γ, which controls the trade-off between sparsity and sensitivity. To document this sensitivity, the network was re-estimated across γ∈ {0.25, 0.50, 0.75, 0.90} both with and without significance-based edge thresholding (Williams and Rast, 2020). Network results are accordingly interpreted as complementary descriptive information about conditional dependencies, not as a structural model of causal propagation.

### 5.8 Measurement precision

Measurement precision was evaluated using model-based reliability to compute standard error of measurement (SEM) and associated confidence intervals (Padilla and Divers, 2016).

### 5.9 Convergent validity sub-study: design and analytic strategy

To complement the internal psychometric and structural analyses, a convergent validity sub-study was conducted in which a subset of the same Italian-speaking community sample (*N* = 76) completed the SDi together with three established external instruments. The external battery comprised the WHO-5 Wellbeing Index (Topp et al., 2015), the Depression Anxiety Stress Scales-21 (DASS-21; Henry and Crawford, 2005), and the Difficulties in Emotion Regulation Scale (DERS; Gratz and Roemer, 2004), selected to span the wellbeing, distress, and emotion-regulation domains most theoretically proximal to psychological integration as conceptualized by the SDi.

Participants completed the full questionnaire battery online with no missing data on the SDi. One participant did not complete the WHO-5 and DASS-21 (yielding *N* = 75 for those scales); all 76 participants completed the DERS. The DERS-36 was administered without item 4, which was inadvertently omitted from the survey instrument; analyses use the remaining 35 items, with the Awareness subscale unaffected by this omission. At *N* = 76, the design has ≥ 0.80 power to detect Pearson correlations of *r* ≥ 0.32 (α = 0.05, two-tailed), which is sufficient for the medium-to-large convergent associations expected at the SDi domain (LV2) level.

Scoring of all instruments followed published algorithms. The WHO-5 was scored as the sum of the five items multiplied by 4 (range 0–100), with higher scores indicating better wellbeing (Topp et al., 2015). The DASS-21 was scored as the sum of seven items per subscale (Depression, Anxiety, Stress) without doubling, in line with current practice for the 21-item form (Henry and Crawford, 2005). The DERS was scored as the mean of available items per subscale and the mean of available items overall, with positively worded items (1, 2, 6, 7, 8, 10, 17, 20, 22, 24, 34) reverse-scored prior to subscale and total computation, following the original scoring algorithm (Gratz and Roemer, 2004); higher DERS scores therefore indicate greater difficulties in emotion regulation. SDi scoring is described in §5.2; for the convergent validity analyses, Disarmonie correlations are reported in their original directionality such that higher Disarmonie scores correspond to greater disharmony.

Convergent validity was evaluated through three complementary analyses. First, Pearson correlations were computed between the four SDi LV2 composites (Total, Isomorfismo, Sé organismico, Disarmonie) and the principal external scales (WHO-5 total, DASS-21 total and subscales, DERS total). Benjamini–Hochberg correction was applied across the 24 primary tests to control the false discovery rate. Second, an exploratory grid of correlations (160 tests) was computed between the SDi LV1 facets and the DERS subscales and DASS components, with BH correction applied across the full grid; this analysis was conducted as a discriminant pattern check, examining whether theoretically proximal facet × subscale pairs yielded substantially stronger correlations than theoretically distal pairs. Third, hierarchical OLS regressions were used to test the incremental contribution of the SDi LV2 blocks beyond Disarmonie alone in predicting WHO-5, DASS Depression, and DERS total scores; for each outcome, Disarmonie was entered at Step 1, Isomorfismo at Step 2, and Sé organismico at Step 3. For the DERS regression, the Emotivo_SE facet replaced Isomorfismo at Step 2 to test the specificity prediction that emotion-regulation difficulties are most proximally associated with the emotional facet of organismic resources.

All correlations are reported with 95% Fisher-z confidence intervals. Hierarchical regressions report unstandardized coefficients and incremental F-tests for nested model comparisons. Skewness and kurtosis were inspected for all primary composites and were within ± 2, supporting the use of Pearson correlations and OLS regression.

### 5.10 Software

Analyses were conducted in R version 4.5.2 (R Core Team, 2025) using the following packages: lavaan version 0.6.21 (Rosseel, 2012) for confirmatory factor analysis and structural equation modeling; semTools version 0.5.8 (Jorgensen et al., 2022) for measurement-invariance testing and reliability indices; psych version 2.6.1 (Revelle, 2025) for exploratory factor analysis, parallel analysis, and sampling-adequacy diagnostics; GPArotation (Bernaards and Jennrich, 2005) for oblimin rotation; qgraph version 1.9.8 (Epskamp et al., 2012) and bootnet version 1.7.1 (Epskamp et al., 2018) for network estimation, regularization, and bootstrap stability assessment; networktools version 1.6.0 (Jones, 2020) for bridge centrality; and car version 3.1.5 (Fox and Weisberg, 2019) for variance inflation factor computation. The full analysis code is available from the corresponding author upon reasonable request.

## 6 Results

### 6.1 Sample characteristics and preliminary associations

The final sample consisted of *N* = 441 participants. The majority identified as women (*n* = 301; 68.3%), followed by men (*n* = 137; 31.1%), with a small proportion identifying as other (*n* = 3; 0.7%). Participants were primarily distributed across Generation X (*n* = 179; 40.6%) and Generation Y (*n* = 144; 32.7%), followed by Baby Boomers (*n* = 82; 18.6%), Generation Z (*n* = 34; 7.7%), the Silent Generation (*n* = 1; 0.2%), and one participant who preferred not to respond (*n* = 1; 0.2%). Percentages are reported to one decimal place; rounding may yield totals between 99.9 and 100.1%.

Mean scores were:

- Organismic resources (SE_G): *M* = 3.73, SD = 0.62
- Integration (ISO_G): *M* = 3.75, SD = 0.53
- Disharmonies (DIS_G): *M* = 2.68, SD = 0.52

Despite significant normality tests (expected in large samples), skewness values indicated only modest deviations from normality.

Preliminary correlations among the three composite scores supported theoretical expectations: organismic resources and integration were strongly positively associated, while disharmonies were negatively associated with both resources and integration (Table 1). All correlations were statistically significant (p < .001), with effect sizes consistent with the hypothesized hierarchical architecture.

**TABLE 1:** Correlation analysis between constructs.

Association	*r*	*p*	95% CI	
SE_G—ISO_G	0.80	<0.001	0.76	0.83
DIS_G—SE_G	–0.55	<0.001	–0.60	–0.47
DIS_G—ISO_G	–0.66	<0.001	–0.70	–0.60

These associations justified proceeding with structural modeling. To address the possibility of multicollinearity at the composite level, variance inflation factors (VIF) were computed by regressing the disharmonies composite (DIS_G) on the resources (SE_G) and integration (ISO_G) composites. Both VIF values were 2.79, with corresponding tolerance values of 0.36 well below the conservative cutoff of 3. The condition number of the predictor correlation matrix was 9.84. These diagnostics indicate that the substantial Q2–Q1 association does not translate into problematic multicollinearity at the composite level.

Because composite-level VIF values can mask issues at the structural-equation level, latent-level VIFs were additionally computed on factor scores extracted from the trimmed structural model (lavPredict). For each endogenous latent variable, the VIF of its structural predictors is reported in Table 2.

**TABLE 2:** Variance inflation factors at the latent level (factor-score-based).

Outcome	Predictors	VIF
DIS_MEN	ISO_MEN	2.33
	ISO_CEX	2.73
	SE_ENE	2.41
DIS_FIS	ISO_CEX	2.49
	SE_ENE	2.56
	SE_EMO	2.22
ISO_MEN	SE_FIS	2.83
	SE_EMO	2.83

**TABLE 3:** Sampling adequacy.

Domain	KMO	Bartlett’s χ^2^(105)	*p*
Integration Q1	0.89	2219.32	<0.001
Organismic resources Q2	0.91	2865.45	<0.001
Disharmonies Q3	0.94	3806.53	<0.001

**TABLE 4:** Retained items and factors for integration Q1.

Factors	Id	Items
Permanence (ISO_PER)	sdi_1	I feel mentally clear and attentive for most of the day.
	sdi_5	I experience a sense of vitality and physical groundedness during the day.
Mental (ISO_MEN)	sdi_2	When facing a difficulty, I consider multiple perspectives before deciding.
	sdi_3	I am aware of my emotions without becoming overwhelmed by them.
	sdi_4	When experiencing emotional distress, I remain connected to myself.
	sdi_10	Under pressure, I choose my responses rather than reacting impulsively.
Internal coherence (ISO_CIN)	sdi_12	When I am in silence, I experience an inner sense of clarity or guidance.
	sdi_13	I think one thing, feel another, and behave in ways that do not align with either.
External coherence (ISO_CEX)	sdi_9	My actions are consistent with what I think and feel.
	sdi_14	I act primarily out of obligation or fear rather than personal conviction.
	sdi_15	I feel empty or disconnected from myself and others.

**TABLE 5:** Retained items and factors for Organismic resources Q2.

Factors	Id	Items
Physical and thinking (SE_FIS)	sdi_16	I feel physically stable and grounded during everyday activities.
	sdi_17	I attend to and respect my body’s signals (e.g., hunger, fatigue, tension).
	sdi_25	My thinking is clear, organized, and flexible in daily life.
	sdi_27	My thoughts are consistent with my deeper feelings.
Energy (SE_ENE)	sdi_18	I experience physical movement and touch as pleasant or energizing.
	sdi_19	I feel physically energized during the morning or peak hours of the day.
	sdi_21	Deep breathing or movement increases my sense of physical vitality.
Emotional (SE_EMO)	sdi_22	I am comfortable experiencing intense or mixed emotions.
	sdi_23	I can distinguish between genuine emotions and reactive responses.
	sdi_24	I express emotions without allowing them to determine my behavior.
Spiritual (SE_SPI)	sdi_28	I feel connected to something greater than myself.
	sdi_29	I experience moments of beauty, awe, or transcendence.
	sdi_30	I have an inner sense of stability that I can access during difficult moments.

All latent-level VIFs fall comfortably below 3, indicating low multicollinearity among structural predictors. Although several Q2–Q1 associations are substantial, the predictors retain sufficient unique variance for stable estimation of structural coefficients.

### 6.2 Exploratory factor analyses

Exploratory factor analyses (EFA) were conducted on the exploratory half (*N* = 220), separately for Q1, Q2, and Q3. Sampling adequacy was strong across all three blocks.

#### 6.2.1 Sampling adequacy

Sampling adequacy was strong across all three Q-blocks, with KMO values exceeding the conventional 0.80 threshold for “meritorious” sampling adequacy and Bartlett’s test of sphericity rejecting the null hypothesis of an identity correlation matrix in all blocks (Table 3). These diagnostics supported the appropriateness of factor analysis for each item set.

#### 6.2.2 Q1—integration

A four-factor solution was retained based on parallel analysis and theoretical coherence. The solution explained 41.9% of total variance.

Four items were removed due to estimation instability (from which sdi_12 identified as ultra-Heywood case identify through uniqueness in the factor analysis). The refined solution demonstrated interpretable and theoretically coherent loadings (Costello and Osborne, 2005).

#### 6.2.3 Q2—organismic resources

A four-factor solution that integrate physic and mental domain domains was retained. The solution explained 53.5% of total variance. Two items were removed due to estimation instability.

#### 6.2.4 Q3—disharmonies

A two-factor solution (Physical and Mental Disharmonies) was retained, explaining 45.2% of total variance. Five items were removed due to cross-loadings or insufficient primary loadings.

The variance explained values are consistent with multidimensional psychological constructs assessed via self-report (Costello and Osborne, 2005). The retained items for each Q-block, organized by latent factor and accompanied by their English translations, are presented in Table 4 (Q1 Integration), 5 (Q2 Organismic resources), and Table 6 (Q3 Disharmonies).

**TABLE 6:** Retained items and factors for disharmonies Q3.

Factors	Id	Items
Physical signals (DIS_FIS)	sdi_31	I feel physically tense or disconnected from my body.
	sdi_32	I neglect my body’s basic needs (e.g., rest, nutrition, movement).
	sdi_33	I have difficulty noticing or interpreting bodily sensations.
	sdi_34	I feel persistently fatigued without a clear physical cause.
Mental and existential (DIS_MEN)	sdi_39	I struggle to understand my emotional state in the present moment.
	sdi_40	I become stuck in repetitive or negative thought patterns.
	sdi_42	My thoughts feel confused or disconnected from the rest of my experience.
	sdi_43	I feel a lack of deeper meaning or direction in my life.
	sdi_44	I feel disconnected from what I consider sacred or personally meaningful.
	sdi_45	I feel completely isolated from others and the world around me.

The refined structure was submitted to confirmatory modeling.

#### 6.2.5 Cross-sample validation

The factor structure derived from the exploratory half (*N* = 220) was submitted to confirmatory factor analysis on the independent confirmatory half (*N* = 221), with the full sample (*N* = 441) reported in parallel for transparency. Table 7 illustrates fit indices highly consistent across the two halves, supporting parameter stability and ruling out sample-specific overfitting.

**TABLE 7:** Cross-sample CFA fit indices per Q-block (WLSMV, scaled).

Block	Sample	χ^2^(df)	*p*	CFI	TLI	RMSEA	RMSEA 90% CI	SRMR
Q1	Confirmatory (*N* = 221)	60.16 (29)	0.001	0.978	0.966	0.070	[0.045, 0.095]	0.050
Q1	Full (*N* = 441)	70.83 (29)	<0.001	0.983	0.974	0.057	[0.040, 0.074]	0.039
Q2	Confirmatory (*N* = 221)	132.56 (59)	<0.001	0.976	0.968	0.075	[0.058, 0.092]	0.052
Q2	Full (*N* = 441)	256.38 (59)	<0.001	0.962	0.949	0.087	[0.076, 0.098]	0.053
Q3	Confirmatory (*N* = 221)	106.06 (34)	<0.001	0.980	0.973	0.098	[0.077, 0.120]	0.047
Q3	Full (*N* = 441)	187.09 (34)	<0.001	0.978	0.970	0.101	[0.087, 0.116]	0.044

Across all three blocks, CFI values exceeded 0.96 in both halves, and the relative pattern of fit indices was preserved across samples. This consistency indicates that the factor structure generalizes beyond the exploratory subsample and is unlikely to reflect sample-specific overfitting. We accordingly retain the full-sample estimates as the primary parameter set throughout the remainder of the Results, with the cross-sample comparison documented here as an overfitting check.

#### 6.2.6 Comparison of competing measurement models

To evaluate whether the hypothesized first-order correlated structure provides the most defensible representation of the SDi item set, three alternative models were estimated for comparison: a unidimensional model, a second-order model, and a bifactor model. Fit indices for all four specifications appear in Table 8.

**TABLE 8:** Fit indices for competing measurement models (full sample, WLSMV).

Model	χ^2^(df)	CFI	TLI	RMSEA	SRMR
1. Unidimensional	3100.51 (495)	0.823	0.812	0.109	0.099
2. First-order correlated	**1291.96 (450)**	**0.943**	**0.933**	**0.065**	**0.053**
3. Second-order	1792.60 (482)	0.911	0.903	0.079	0.069
4. Bifactor	1909.99 (462)	0.977	0.973	0.084	0.073

Bold values indicate the retained measurement model (first-order correlated structure), selected as the most defensible specification.

The unidimensional model fit poorly on all indices (CFI = 0.82; RMSEA = 0.11; SRMR = 0.10), confirming that the SDi cannot be reduced to a single general factor. The first-order correlated model fit substantially better than the unidimensional specification [scaled Δχ^2^(45) = 1017.4, *p* < 0.001], and also outperformed the second-order specification [scaled Δχ^2^(32) = 409.9, *p* < 0.001], indicating that the higher-order constraint imposed by the second-order model (namely, that all between-factor covariances are explained by three superordinate latent variables) is not consistent with the data. Although the bifactor model produced the highest CFI value, it failed to converge cleanly under WLSMV (with warnings concerning a non-invertible information matrix and a non-positive-definite latent covariance matrix), indicating that the parameter estimates and standard errors from this specification are not statistically reliable. Furthermore, recent methodological work has highlighted the tendency of bifactor models to overfit by absorbing residual covariance into the general factor, producing inflated CFI values without corresponding theoretical interpretability (Bonifay et al., 2017).

Taken together, the comparisons converge on the first-order correlated structure as the most defensible specification: it offers substantially better fit than the unidimensional and second-order alternatives, while avoiding the inferential instability of the bifactor model. We therefore retain this structure as the measurement model underlying all subsequent structural analyses.

### 6.3 Measurement model evaluation

#### 6.3.1 Measurement model

The first-order correlated measurement model demonstrated good approximate fit, scaled χ^2^(450) = 1291.96, *p* < 0.001, CFI = 0.943, TLI = 0.933, RMSEA = 0.065 (90% CI [0.060, 0.070]), SRMR = 0.053. CFI and TLI exceeded the 0.90 threshold for adequate fit and approached the 0.95 benchmark for excellent fit, while RMSEA and SRMR were within their respective acceptable ranges (Hu and Bentler, 1999). These indices represent a substantial improvement over the previous estimation strategy and support the adequacy of the measurement structure for ordinal indicators.

All indicators loaded significantly on their intended latent constructs (*p* < 0.001). Standardized factor loadings were generally strong across constructs. As is common for ordinal estimation, all loadings were statistically significant and theoretically coherent. Indicator-level R^2^ values ranged from approximately 0.23–0.80, supporting satisfactory indicator reliability across the measurement structure.

Composite reliability (ω) estimates demonstrated strong internal consistency across all constructs, ranging from 0.68 to 0.92, with all but one construct (ISO_PER ω = 0.68) exceeding the 0.70 threshold (Q2: SE_FIS = 0.80, SE_ENE = 0.77, SE_EMO = 0.82, SE_SPI = 0.89; Q1: ISO_PER = 0.68, ISO_MEN = 0.81, ISO_CIN = 0.91, ISO_CEX = 0.73; Q3: DIS_FIS = 0.84, DIS_MEN = 0.92). Average Variance Extracted (AVE) values were satisfactory for the majority of constructs (0.51–0.85), with only ISO_CEX (0.48) and ISO_PER (0.52, at the threshold) approaching the 0.50 boundary. The combination of strong composite reliability and acceptable AVE supports adequate convergent validity across the measurement model.

Discriminant validity was evaluated using the Heterotrait–Monotrait ratio (HTMT). All HTMT values fell below the conservative 0.85 cutoff (Henseler et al., 2015; Cheung et al., 2024), supporting the empirical distinctiveness of the constructs. Among the higher pairwise HTMT values, ISO_CEX showed elevated but acceptable associations with DIS_FIS (HTMT = 0.80) and DIS_MEN (HTMT = 0.84), and the two disharmony constructs were closely related (HTMT = 0.82). These patterns are theoretically expected given that external coherence is conceptualized as the proximal regulatory mediator buffering against both disharmony domains, while DIS_FIS and DIS_MEN reflect parallel manifestations of systemic dysregulation. Despite their theoretical proximity, all constructs remained empirically distinguishable. Standardized factor loadings, standard errors, and indicator reliabilities for the full measurement model are presented in Table 9.

**TABLE 9:** Standardized Factor Loadings and Indicator Reliability (WLSMV estimation, polychoric correlations).

Construct	Item	λ	SE	*p*	R^2^
SE_FIS	sdi_16	0.785	0.025	0	0.616
SE_FIS	sdi_17	0.728	0.028	0	0.53
SE_FIS	sdi_25	0.799	0.023	0	0.638
SE_FIS	sdi_27	0.486	0.04	0	0.236
SE_ENE	sdi_18	0.701	0.034	0	0.492
SE_ENE	sdi_19	0.796	0.032	0	0.634
SE_ENE	sdi_21	0.675	0.034	0	0.456
SE_EMO	sdi_22	0.753	0.035	0	0.568
SE_EMO	sdi_23	0.732	0.03	0	0.535
SE_EMO	sdi_24	0.828	0.024	0	0.685
SE_SPI	sdi_28	0.803	0.025	0	0.645
SE_SPI	sdi_29	0.854	0.021	0	0.729
SE_SPI	sdi_30	0.893	0.022	0	0.798
ISO_PER	sdi_1	0.658	0.037	0	0.433
ISO_PER	sdi_5	0.788	0.028	0	0.621
ISO_MEN	sdi_2	0.659	0.037	0	0.434
ISO_MEN	sdi_3	0.802	0.025	0	0.644
ISO_MEN	sdi_4	0.836	0.023	0	0.698
ISO_CIN	sdi_12	1.201	0.071	0	1.443
ISO_CIN	sdi_13	0.537	0.039	0	0.288
ISO_CEX	sdi_9	0.731	0.032	0	0.535
ISO_CEX	sdi_14	0.565	0.036	0	0.32
ISO_CEX	sdi_15	0.74	0.032	0	0.548
DIS_FIS	sdi_31	0.697	0.028	0	0.486
DIS_FIS	sdi_32	0.733	0.026	0	0.537
DIS_FIS	sdi_33	0.665	0.031	0	0.442
DIS_FIS	sdi_34	0.897	0.02	0	0.804
DIS_MEN	sdi_39	0.719	0.029	0	0.518
DIS_MEN	sdi_40	0.778	0.022	0	0.605
DIS_MEN	sdi_42	0.89	0.015	0	0.791
DIS_MEN	sdi_43	0.894	0.014	0	0.799

*p*-values are provided for freely estimated loadings. Marker indicators (first item per factor, fixed for identification) have no associated standard error and *p*-value.

#### 6.3.2 Structural model

Before reporting the structural results, we make explicit the steps taken to constrain interpretive flexibility. The directional hypothesis blocks H1–H5 (§4.6) were specified prior to model estimation, and the construct definitions used throughout this section, including the interpretation of ISO_CEX as external coherence (alignment between thought, feeling, and action), derive from the theoretical architecture in §4 rather than from *post-hoc* relabeling. The trimming criterion (| β| ≥ 0.10 and *p* < 0.05 in the full model) was specified prior to inspecting individual paths and was applied uniformly to all directional structural paths. The split-sample design (§5.4) further constrains the risk of structure-fitting on the same data used to derive it, and the comparison against unidimensional, second-order, and bifactor specifications (§6.2.6) tests the first-order correlated structure against alternatives on objective fit grounds rather than theoretical preference. Within these constraints, the suppression pattern observed for SE_EMO → DIS_FIS emerged in the data and is reported as the empirical resolution of the only exploratory hypothesis in the model (H4c); we mark this distinction transparently rather than absorb the result into the confirmatory frame.

Following evaluation of the full structural specification [scaled χ^2^(402) = 1477.26, CFI = 0.922, RMSEA = 0.078, SRMR = 0.063], a reduced model retaining only statistically robust paths was estimated. This model preserved the theoretically central hierarchical structure while eliminating non-significant cross-domain paths. Model fit remained acceptable, scaled χ^2^(416) = 1458.06, *p* < 0.001, CFI = 0.924, TLI = 0.915, RMSEA = 0.075 (90% CI ≈ [0.070, 0.080]), SRMR = 0.065, indicating that parsimony did not degrade approximate fit and confirming the adequacy of the trimmed structural specification. We note that within the SDi framework, ISO_CEX denotes *external coherence* (alignment between thought, feeling, and action) rather than existential isolation; consequently, negative associations between ISO_CEX and the disharmony outcomes (i.e., higher external coherence corresponding to lower disharmony) are theoretically expected.

#### 6.3.3 Direct structural effects

At the structural level, the following pathways were statistically significant in the trimmed model. Resource → integration paths (Q2 → Q1):

- SE_FIS → ISO_MEN (β = 0.41, *p* < 0.001)—supports H1a
- SE_EMO → ISO_MEN (β = .47, *p* < 0.001)—supports H1b
- SE_FIS → ISO_CEX (β = 0.82, *p* < 0.001)—supports H1c
- SE_SPI → ISO_CIN (β = 0.68, *p* < 0.001)—supports H1d

Integration → dysregulation paths (Q1 → Q3):

- ISO_CEX → DIS_FIS (β = -0.65, *p* < 0.001)—supports H2a
- ISO_CEX → DIS_MEN (β = -0.68, *p* < 0.001)—supports H2a
- ISO_MEN → DIS_MEN (β = -0.18, *p* = 0.001)—supports H2b

Direct resource → dysregulation paths (Q4):

- SE_ENE → DIS_FIS (β = -0.42, *p* < 0.001)—supports H4a
- SE_ENE → DIS_MEN (β = -0.07, *p* = 0.268)—does not support H4b
- SE_EMO → DIS_FIS (β = +.21, *p* < 0.001)—exploratory path (H4c); cfr. Suppression Analysis below.

External coherence (ISO_CEX) emerged as the dominant proximal predictor of both physical and mental disharmonies, with large standardized effects. This finding structurally positions enacted congruence (alignment between thought, feeling, and action) as the most immediate buffer against dysregulation signals. Energetic organization (SE_ENE) retained a direct negative association with physical disharmony, indicating that vitality-related processes exert effects not fully mediated by integration. The direct association of SE_ENE with mental disharmony was not statistically significant once integration and other resources were modeled simultaneously. The substantial standardized association between SE_FIS and ISO_CEX (β = 0.82) reflects the close theoretical coupling of physical–cognitive grounding and behavioral enactment within the SDi architecture; the constructs nevertheless remain empirically distinguishable, as confirmed by HTMT values below 0.85 and latent-level VIFs below 3.

The model explained substantial variance:

- DIS_FIS: *R*^2^ = 0.71
- DIS_MEN: *R*^2^ = 0.72
- ISO_MEN: *R*^2^ = 0.66
- ISO_CEX: *R*^2^ = 0.67
- ISO_CIN: *R*^2^ = 0.46

Thus, the architecture demonstrated strong explanatory coherence at the regulatory level. The pattern of variance explained (highest for the dysregulation outcomes, intermediate for the integration mediators, and lowest for internal coherence) is consistent with the theoretical expectation that disharmonies represent the most strongly determined endpoint of the hierarchical pathway, while integration domains operate as partially saturated mediators reflecting genuine but incomplete integrative coupling.

#### 6.3.4 Suppression analysis: SE_EMO → DIS_FIS

The SE_EMO → DIS_FIS path is the only structural path in the model for which no directional prediction was advanced prior to estimation: H4c (§4.6, Block 4) was explicitly registered as exploratory because the literature does not converge on a single expected sign, with both protective and inconsistent-mediation patterns considered theoretically defensible. The decomposition reported below is therefore the empirical resolution of a pre-registered exploratory hypothesis rather than a *post-hoc* explanation of an unexpected coefficient.

The positive direct association between emotional organization (SE_EMO) and physical disharmony (DIS_FIS) was investigated through a formal decomposition of the total effect into direct and indirect components. A model was estimated in which SE_EMO had both a direct path to DIS_FIS and an indirect path via ISO_MEN, allowing the relative contributions of each to be quantified.

The decomposition yielded three findings: (a) the direct effect was positive (β = +0.21); (b) the indirect effect via ISO_MEN was negative (β = -0.07); and (c) the total effect was small and positive (β = +0.14). The opposite signs of the direct and indirect components are the diagnostic signature of an inconsistent-mediation or *suppression* pattern (MacKinnon et al., 2000). In this configuration, two opposing pathways operate simultaneously: emotional organization exerts a protective indirect effect on physical disharmony through enhanced mental integration, while a residual direct path remains positive after the integrative pathway is accounted for.

Theoretically, this pattern is interpretable rather than anomalous. Emotional differentiation has been argued to support reflective regulation (Barrett, 2017), which is the protective channel captured by the indirect effect via ISO_MEN. At the same time, heightened emotional awareness can plausibly increase interoceptive sensitivity to somatic signals, amplifying the perception of bodily strain (Khalsa et al., 2018). The two channels operate in opposite directions and partially cancel out in the total effect, yielding the small positive net association observed. The path is therefore not theoretically inconsistent; rather, it reflects the dual valence of emotional differentiation as both a reflective and a sensorial capacity, which is a substantive finding rather than a statistical artifact.

The exploratory designation of this path (H4c) reflects the absence of a directional prediction in advance: both protective and inconsistent-mediation patterns were considered theoretically defensible. The empirical result is most consistent with the latter. We acknowledge that the inconsistent-mediation interpretation requires confirmation on independent samples before substantive theoretical claims; the present finding establishes the pattern empirically and locates it within a pre-specified exploratory frame.

### 6.4 Bootstrapped mediation effects

To evaluate indirect pathways, nonparametric bootstrapping (5,000 resamples; bias-corrected confidence intervals) was conducted.

#### 6.4.1 Significant indirect effects

Two robust mediation pathways emerged, supporting H3:

- SE_FIS → ISO_CEX → DIS_FIS: β = –0.49, 95% CI [–0.75, –0.39] (BCa bootstrap, 5,000 resamples)
- SE_FIS → ISO_CEX → DIS_MEN: β = –0.58, 95% CI [–0.70, –0.40] (BCa bootstrap, 5,000 resamples)

These effects indicate that the association between SE_FIS organization and both forms of disharmony is largely transmitted through ISO_CEX.

Additionally:

3. SE_EMO → ISO_MEN → DIS_MEN

4. β = –0.05, 95% CI [–0.11, –0.002]

Although small in magnitude, this indirect pathway reached statistical significance, indicating that SE_EMO contributes to reduced DIS_MEN through enhanced ISO_MEN.

#### 6.4.2 Total effects

Total effects (direct + indirect, where applicable) confirmed the structural prominence of physical–cognitive organization within the trimmed model:

- SE_FIS → DIS_FIS (total): β = –0.49
- SE_FIS → DIS_MEN (total): β = –0.58
- SE_ENE → DIS_FIS (direct only): β = –0.42
- SE_ENE → DIS_MEN (direct only): β = –0.07, n.s.

The full set of total effects, with bootstrap 95% confidence intervals, is reported in Table 12.

The absence of a significant total indirect effect for SE_ENE reinforces the interpretation that energetic vitality operates partly outside the integrative mediation chain.

#### 6.4.3 Partial vs. full mediation

Comparison with a constrained full-mediation model (in which the direct paths from organismic resources to disharmonies were fixed to zero) significantly degraded model fit, supporting the retention of the partial-mediation specification reported above. Direct effects of energetic organization (SE_ENE → DIS_FIS) and the exploratory direct path (SE_EMO → DIS_FIS) provide effects that cannot be reduced to indirect transmission through integration mediators.

#### 6.4.4 Structural summary

**FIGURE 1:**
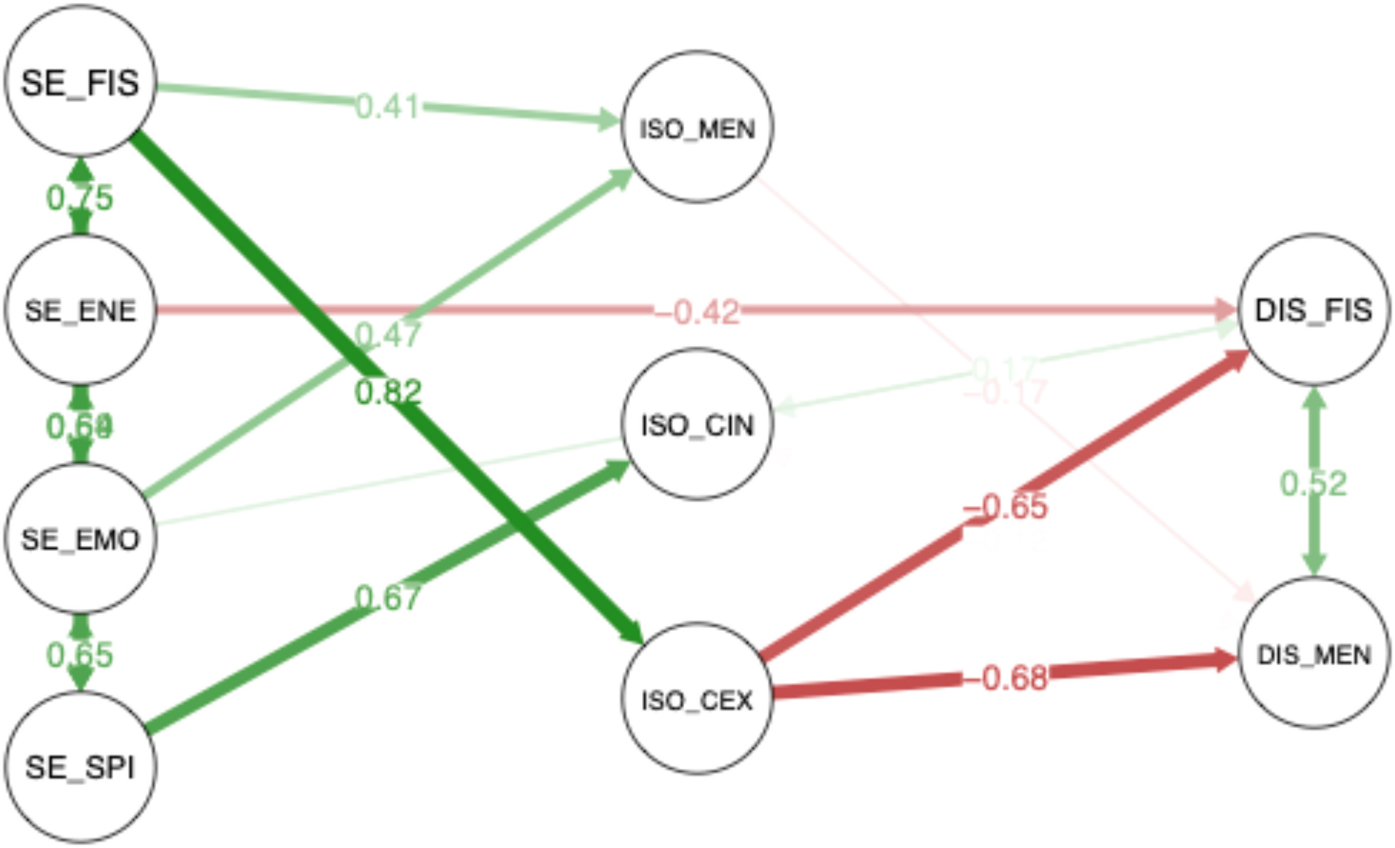
Trimmed structural model of the SDi (standardized solution, WLSMV). Only paths with | β| ≥ 0.10 and *p* < 0.05 are displayed. Edge thickness encodes magnitude; color encodes sign (green = positive, red = negative); transparency encodes residual variance. Latent constructs are shown as ellipses; measurement indicators are omitted for clarity. Curved double-headed arrows denote covariances; the residual covariance between DIS_FIS and DIS_MEN (H5) is shown explicitly. The path SE_EMO → DIS_FIS is exploratory (H4c) and is interpreted in the Suppression Analysis subsection.

The full pattern of significant standardized paths in the trimmed structural model is depicted in Figure 1. The figure follows the visual conventions recommended for SEM diagrams (Edge thickness encodes magnitude; color encodes sign; transparency encodes residual variance), with paths below | β| = 0.10 omitted to support clear interpretation.

Collectively, the final structural model suggests the following pattern of cross-sectional associations:

1. Organismic resources are differentially associated with specific integration domains.
2. External coherence is the principal proximal correlate of both somatic and cognitive–existential disharmony, with the largest standardized association of any predictor in the system.
3. Energetic vitality retains a direct association with somatic disharmony that is not statistically accounted for by the integration mediators.
4. Disharmonies exhibit both hierarchical organization (with integration as proximal correlate) and conditional interdependence among themselves (examined in the network analysis below).

The resulting pattern is consistent with the theoretical architecture of a hierarchically organized, multi-level system in which structural resources are associated with integration, integration is associated with reduced disharmony, and disharmonies are conditionally interdependent. The cross-sectional design supports inference about associations among these levels but does not establish their temporal sequence.

Direct structural effects, including standardized coefficients, standard errors, *p*-values, bootstrap-based 95% confidence intervals, and R^2^ values for each endogenous latent variable, are reported in Table 10.

**TABLE 10:** Structural model direct effect—bootstrap 95% CI (5,000 resamples).

Outcome	Predictor	Standardized β	SE	*P*	CI_lower	CI_upper	*R* ^2^
SO_MEN	SE_FIS	0.420	0.092	< 0.001	0.168	0.528	0.643
ISO_MEN	SE_EMO	0.449	0.077	< 0.001	0.194	0.494	0.643
ISO_CIN	SE_SPI	0.615	0.053	< 0.001	0.412	0.619	0.378
ISO_CEX	SE_FIS	0.776	0.101	< 0.001	0.421	0.817	0.603
DIS_FIS	ISO_CEX	–0.636	0.185	< 0.001	-1.286	–0.553	0.751
DIS_MEN	ISO_MEN	–0.115	0.072	0.072	–0.283	0.001	0.732
DIS_MEN	ISO_CEX	–0.687	0.160	< 0.001	–1.153	–0.536	0.732
DIS_FIS	SE_ENE	–0.450	0.114	< 0.001	–0.794	–0.351	0.751
DIS_FIS	SE_EMO	0.193	0.069	0.005	0.062	0.327	0.751
DIS_MEN	SE_ENE	–0.145	0.072	0.043	–0.291	–0.003	0.732

Indirect (mediated) effects from organismic resources to disharmonies, transmitted through the integration domains, are reported in Table 11 with bootstrap 95% confidence intervals (5,000 resamples, BCa method).

**TABLE 11:** Structural model indirect effect—bootstrap 95% CI (5,000 resamples).

Outcome	Predictor	Pathway	Standardized. β	SE	*P*	CI_lower	CI_upper
DIS_FIS	SE_FIS	Via ISO_CEX	–0.494	0.090	< 0.001	–0.742	–0.382
DIS_MEN	SE_FIS	Via ISO_CEX (+ ISO_MEN chain)	–0.582	0.076	< 0.001	–0.702	–0.399
DIS_MEN	SE_EMO	Via ISO_MEN	–0.052	0.026	0.102*	–0.110	–0.002

The BCa bootstrap 95% CI excludes zero, indicating a statistically significant indirect effect; the bootstrap confidence interval is the inferential criterion for indirect effects, whereas the accompanying *p*-value is Delta-method based.

Total effects (direct plus indirect) from organismic resources to disharmonies are reported in Table 12, providing a summary of the overall associations within the trimmed structural model.

**TABLE 12:** Structural model total effect—bootstrap 95% CI (5,000 resamples)

Outcome	Predictor	Standardized β	SE	*p*	CI_lower	CI_upper
DIS_FIS	SE_FIS	–0.494	0.090	< 0.001	–0.742	–0.382
DIS_MEN	SE_FIS	–0.582	0.076	< 0.001	–0.702	–0.399
DIS_FIS	SE_ENE	–0.450	0.110	< 0.001	–0.783	–0.346
DIS_MEN	SE_ENE	–0.145	0.072	0.040	–0.288	–0.005
DIS_MEN	SE_EMO	–0.052	0.026	0.102	–0.110	–0.002

### 6.5 Supplementary analyses: gender measurement invariance

Given the gender distribution of the sample (68% women; 32% men), multi-group invariance across gender was tested using the primary WLSMV estimator on a polychoric correlation matrix and, for comparison, the supplementary MLR estimator. Five items (sdi_3, sdi_4, sdi_9, sdi_16, sdi_27) had empty bottom response categories in one gender group; for the WLSMV invariance analysis only, the bottom two categories were collapsed uniformly across groups (Liu et al., 2017; Wu and Estabrook, 2016). The substantive CFA and structural results reported elsewhere in the manuscript used the original five-point scale.

#### 6.5.1 Configural invariance

The configural model showed acceptable fit under both estimators (WLSMV: CFI = 0.937, RMSEA = 0.072, SRMR = 0.07; MLR: CFI = 0.906, RMSEA = 0.057, SRMR = 0.061), supporting equivalent factor structure across women and men.

#### 6.5.2 Metric invariance

Under WLSMV, constraining loadings and thresholds did not degrade fit (CFI = 0.943, RMSEA = 0.065; ΔCFI = +0.006, ΔRMSEA = -0.007). Under MLR, the comparable model also showed no significant degradation, Δχ^2^(21) = 17.61, *p* = 0.67. Metric invariance is therefore supported under both estimators, indicating that items relate to their latent constructs equivalently across gender.

#### 6.5.3 Scalar invariance

The two estimators converge on a more nuanced picture. Under WLSMV, additionally constraining intercepts produced negligible changes in fit (CFI = 0.944, RMSEA = 0.064; ΔCFI = +0.001, ΔRMSEA = -0.001), well within the Chen (2007) thresholds for invariance. Under MLR, the same constraint produced a statistically significant χ^2^ difference [Δχ^2^(22) = 41.69, *p* = 0.007]. The divergence between fit-index changes and the χ^2^ difference test in large samples is well documented (Cheung and Rensvold, 2002): the χ^2^ test is sensitive to sample size and can flag practically negligible differences as statistically significant. Following current methodological recommendations (Chen, 2007; Cheung and Rensvold, 2002; Putnick and Bornstein, 2016), we report fit-index changes as the primary criterion under WLSMV.

#### 6.5.4 Adopted conclusion: partial scalar invariance

The lavTestScore diagnostic on the WLSMV scalar model identified a small number of intercept/threshold constraints that contributed to misfit (X^2^ values 8.9–10.8 for the largest contributors). Following Byrne et al. (1989), we adopt a *partial scalar invariance* conclusion: scalar invariance is supported as a global property of the measurement model under fit-index criteria, but a small number of items show modest intercept differences across gender. Structural-coefficient comparisons across gender are therefore warranted on the strength of full metric invariance; latent-mean comparisons are also warranted, but should free the non-invariant intercepts when conducted (Putnick and Bornstein, 2016). For the present manuscript, structural analyses do not depend on latent-mean equivalence, and the partial scalar finding does not affect the interpretation of the structural model.

### 6.6 Network analysis of disharmonies

To further investigate the structural properties of Disharmonies (Q3) beyond the constraints of reflective latent modeling, a network analysis was conducted. In this framework, items are conceptualized as mutually interacting regulatory signals rather than reflective indicators of a single higher-order latent trait. Edges represent regularized partial correlations controlling for all other nodes in the network. As detailed below, network results are interpreted descriptively: edges describe conditional dependencies in cross-sectional data and do not support causal or directional inference.

To assess structural robustness, nonparametric case-dropping bootstrap procedures (2000 resamples) were performed.

**FIGURE 2:**
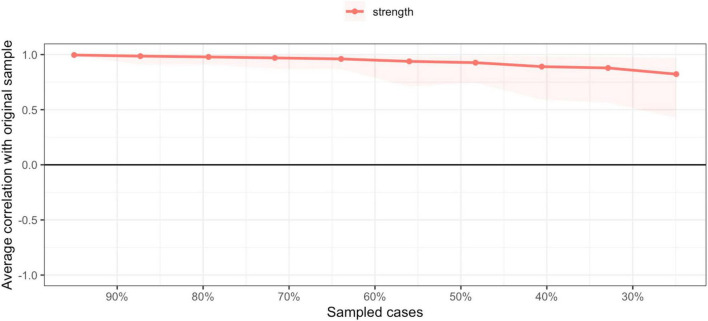
Correlation stability plot of the disharmonies network.

The network showed strong structural stability under case-dropping bootstrap, with a Correlation Stability coefficient of 0.67 for expected influence and 0.52 for node strength (above the 0.50 threshold for stable estimation (Epskamp et al., 2018). The CS plot in Figure 2 visualizes the average correlation between the original centrality estimates and those from samples with progressively larger proportions of cases removed; values above 0.7 across the full range of subsample sizes indicate that the centrality estimates remain stable under substantial subsampling.

**FIGURE 3:**
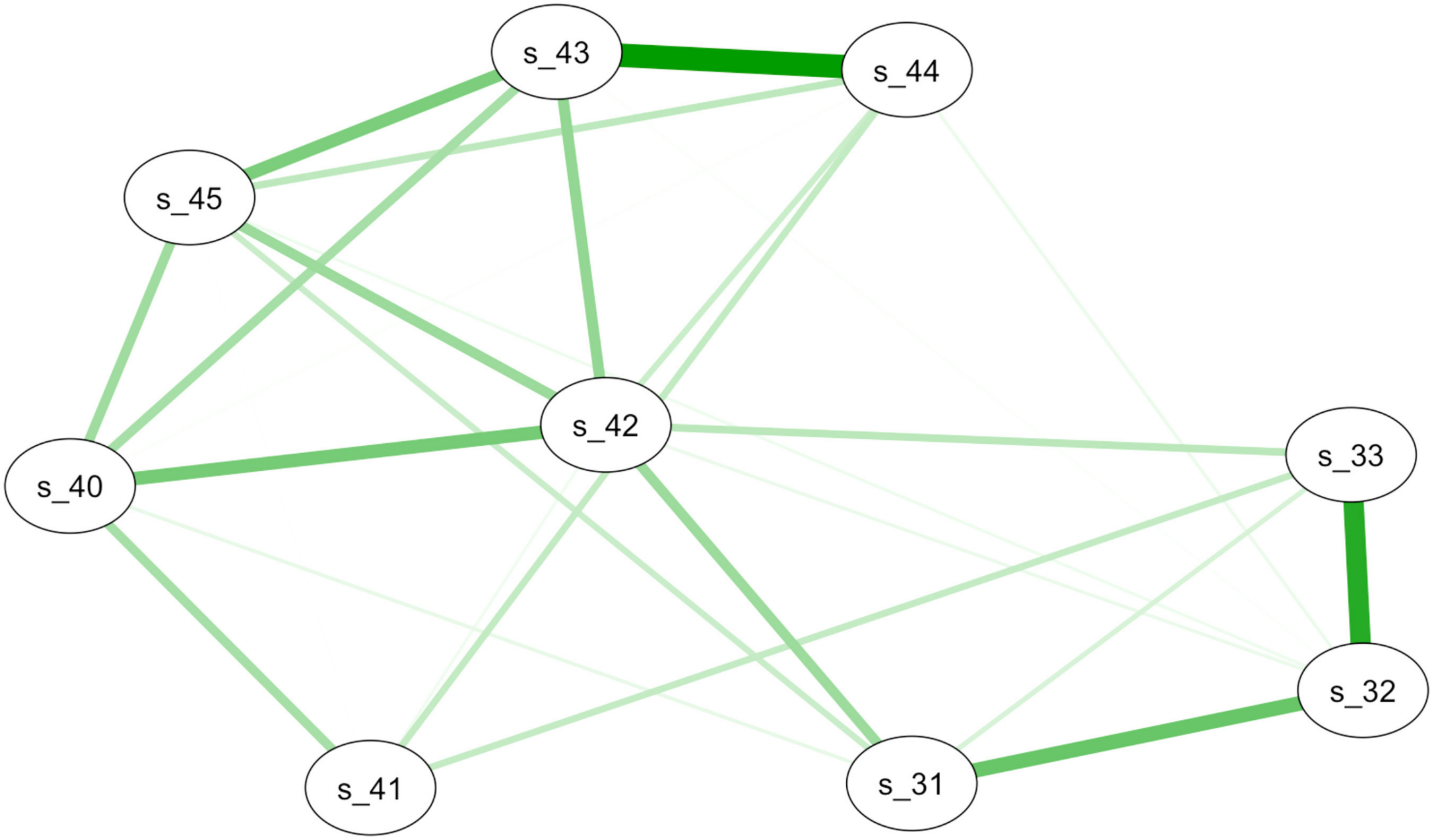
Regularized partial correlation network of Disharmonies (Q3). Edge thickness indicates strength of conditional associations. Bootstrapped stability coefficients indicate strong robustness (CS_strength_ = 0.52; _CSexpected_ _influence_ = 0.67).

The regularized partial correlation network of Disharmonies (Q3), depicted in Figure 3, shows dense interconnectivity within both Physical and Mental communities of items, with several inter-community edges. Edge thickness indicates the strength of conditional associations after controlling for all other items in the network. The visual structure is consistent with a description of dysregulation indicators as conditionally interdependent rather than reflective outputs of a single latent dysregulation factor.

To further investigate the interconnectivity between the Physical and Mental constructs of Q3, bridge centrality was evaluated (Jones et al., 2018). While node strength identifies the most influential items within the entire system, bridge centrality highlights items conditionally associated with both communities of nodes. The strongest bridge in the present network was sdi_34 (“I feel persistently fatigued without a clear physical cause”; bridge strength = 0.47), followed by sdi_42 (“My thoughts feel confused or disconnected from the rest of my experience”; bridge strength = 0.30) and sdi_39 (“I struggle to understand my emotional state in the present moment”; bridge strength = 0.24). The remaining items showed lower bridge values (range 0.11–0.24). The bridge ordering is consistent with the theoretical interpretation that persistent unexplained fatigue and cognitive disconnection function as conditional gateways linking somatic and cognitive–existential dysregulation in the Q3 indicator set, while precluding any causal interpretation of these associations.

The network exhibited dense local interconnectivity, with edges spanning all pairs of items within both Physical and Mental communities and several cross-community edges. To document the robustness of this structure to the regularization choice, the network was re-estimated across γ∈ {0.25, 0.50, 0.75, 0.90}, both with and without significance-based edge thresholding (Williams and Rast, 2020). Edge counts were stable across the full range: 33 edges without thresholding and 23 edges under thresholding, irrespective of γ. This invariance indicates that the dense connectivity observed at γ = 0.50 is not an artifact of the regularization choice but reflects substantial conditional interdependence among the Q3 indicators. Bridge node identification was also stable: sdi_34 emerged as the dominant bridge across all γ values tested.

The mediation findings of the structural model and the network analysis of disharmonies are descriptively consistent. The dominant role of external coherence in predicting both disharmony domains in the structural model is paralleled in the network by the dense conditional interconnections among Q3 indicators across both somatic and cognitive–existential communities. The identification of sdi_34 (persistent unexplained fatigue) as the strongest bridge, with sdi_42 (cognitive disconnection) and sdi_39 (difficulty understanding present emotional state) as secondary bridges, is consistent with the broader theoretical reading that disharmonies form a distributed regulatory system in which somatic and cognitive–existential signals are conditionally linked. We emphasize, however, that this consistency between the SEM and network results is interpretive convergence, not a causal claim: the cross-sectional design does not permit inference about the temporal direction or generative mechanism of these associations.

### 6.7 Measurement precision

Measurement precision indices were calculated for each Q-block by combining composite reliability (ω) with construct-level standard error of measurement (SEM), with the resulting 95% confidence interval representing the expected range of repeated-measure variation for an individual respondent (Padilla and Divers, 2016). Results are presented in Table 13.

**TABLE 13:** Measurement precision—SEM computed under congeneric reliability assumptions.

SDi block	ω	SEM	95% CI (± 1.96 × SEM)
Organismic resources Q2	0.776	0.29	± 0.58
Integration Q1	0.779	0.25	± 0.49
Disharmonies Q3	0.892	0.17	± 0.34

Disharmonies (Q3) demonstrated the highest measurement precision (SEM = 0.17, 95% CI ± 0.34), followed by Integration (Q1; SEM = 0.25, 95% CI ± 0.49) and Organismic resources (Q2; SEM = 0.29, 95% CI ± 0.58). The relatively higher precision for the Disharmonies block is consistent with its higher composite reliability (ω = 0.89) and its larger number of indicators per latent factor.

### 6.8 Convergent evidence against WHO-5, DASS-21, and DERS

#### 6.8.1 Descriptive statistics for external scales

Descriptive statistics for the three external instruments are presented in Table 14. The WHO-5 mean (*M* = 61.6, SD = 18.2) is broadly consistent with non-clinical adult community norms (Topp et al., 2015), and the DASS-21 subscale means fall within the normal-to-mild range relative to published cut-offs when the seven-item sums are doubled to compare with the DASS-42 reference distribution (Henry and Crawford, 2005). The DERS subscale means show the typical pattern in non-clinical samples, with awareness, strategies, and clarity at lower (more adaptive) levels and nonacceptance at moderate levels. Skewness and kurtosis values were within ± 2 for all primary composites, supporting the use of Pearson correlations.

**TABLE 14:** Descriptive statistics for external benchmark scales (*N* = 75–76).

Scale/Construct	*N*	*M*	SD	Min	Max
*WHO-5 (total, 0–100)*	*75*	61.60	*18.20*	*16*	*92*
*DASS-21 total*	*75*	*18.40*	*9.76*	*1*	*47*
DASS depression	75	5.83	4.27	0	19
DASS anxiety	75	5.51	3.10	0	13
DASS stress	75	7.05	3.70	1	18
*DERS total (mean of 32 items)*	*76*	*2.30*	*0.40*	*1.69*	*3.75*
DERS nonacceptance	76	2.73	0.44	1.83	5.00
DERS goals	76	2.59	0.51	1.25	4.00
DERS impulse	76	2.50	0.39	1.67	4.33
DERS awareness	76	2.09	0.67	1.00	4.00
DERS strategies	76	1.97	0.56	1.17	4.33
DERS clarity	76	1.95	0.92	1.00	4.75

Italicized rows are subscales of the preceding total scale.

#### 6.8.2 Primary convergent correlations: SDi LV2 domains × external scales

Pearson correlations between the four SDi LV2 composites (Total, Isomorfismo, Sé organismico, Disarmonie) and the principal external scales are reported in Table 15. All 24 primary correlations were statistically significant after Benjamini–Hochberg correction for multiple testing, and all 95% confidence intervals excluded zero in the predicted direction.

**TABLE 15:** Pearson correlations between SDi LV2 composites and external benchmark scales (*N* = 75–76).

SDi domain	External scale	*N*	*r*	95% CI	*p* (BH)
SDi total	WHO-5	75	0.82	[0.73, 0.88]	< 0.001
SDi total	DASS-21 total	75	–0.67	[–0.78, –0.53]	< 0.001
SDi total	DASS depression	75	–0.74	[–0.83, –0.61]	< 0.001
SDi total	DASS anxiety	75	–0.44	[–0.60, –0.23]	< 0.001
SDi total	DASS stress	75	–0.56	[–0.70, –0.38]	< 0.001
SDi total	DERS total	76	–0.70	[–0.80, –0.56]	< 0.001
Isomorfismo (Q1)	WHO-5	75	0.78	[0.67, 0.85]	< 0.001
Isomorfismo (Q1)	DASS-21 total	75	–0.63	[–0.75, –0.47]	< 0.001
Isomorfismo (Q1)	DASS depression	75	–0.72	[–0.81, –0.59]	< 0.001
Isomorfismo (Q1)	DASS anxiety	75	–0.39	[–0.57, –0.18]	< 0.001
Isomorfismo (Q1)	DASS stress	75	–0.51	[–0.66, –0.32]	< 0.001
Isomorfismo (Q1)	DERS total	76	–0.68	[–0.78, –0.54]	< 0.001
Sé organismico (Q2)	WHO-5	75	0.75	[0.63, 0.83]	< 0.001
Sé organismico (Q2)	DASS-21 total	75	–0.58	[–0.71, –0.41]	< 0.001
Sé organismico (Q2)	DASS depression	75	–0.62	[–0.74, –0.46]	< 0.001
Sé organismico (Q2)	DASS anxiety	75	–0.39	[–0.57, –0.18]	< 0.001
Sé organismico (Q2)	DASS stress	75	–0.48	[–0.64, –0.29]	< 0.001
Sé organismico (Q2)	DERS total	76	-0.63	[–0.75, –0.47]	< 0.001
Disarmonie (Q3)	WHO-5	75	–0.80	[–0.87, -0.69]	< 0.001
Disarmonie (Q3)	DASS-21 total	75	0.70	[0.56, 0.80]	< 0.001
Disarmonie (Q3)	DASS depression	75	0.75	[0.63, 0.83]	< 0.001
Disarmonie (Q3)	DASS anxiety	75	0.46	[0.26, 0.62]	< 0.001
Disarmonie (Q3)	DASS stress	75	0.59	[0.41, 0.72]	< 0.001
Disarmonie (Q3)	DERS total	76	0.66	[0.52, 0.77]	< 0.001

*p*-values are Benjamini–Hochberg adjusted across the 24 primary tests. 95% CIs are unadjusted Fisher-z intervals. Disarmonie correlations are reported in original directionality (higher = greater disharmony).

Three features of the pattern support the SDi’s convergent validity. First, the SDi total score correlated strongly with the WHO-5 (*r* = 0.82) and substantially with the DASS-21 total (*r* = -0.67) and DERS total (*r* = -0.70), supporting global convergence with established wellbeing, distress, and emotion-regulation benchmarks. Second, the four LV2 composites showed convergent associations of comparable magnitude with the external scales, with Disarmonie and Isomorfismo emerging as the strongest individual predictors of the WHO-5 (|*r*| = 0.80 and 0.78, respectively) and Disarmonie as the strongest predictor of DASS Depression (*r* = 0.75). This pattern is consistent with the structural model reported in §6.3, in which the three Q-blocks index closely related but distinguishable facets of a hierarchically organized integration system.

Third, the strongest external correlate of every SDi composite was the WHO-5, followed by DASS Depression and DERS total, with DASS Anxiety yielding the weakest associations across all four LV2 composites (|*r*| range = 0.39–0.46). This ordering is theoretically coherent: wellbeing and depression are global indicators of organismic coherence and demoralization respectively, both of which are theorized as proximal correlates of integration; anxiety, by contrast, includes a strong autonomic-arousal component that is less directly indexed by the SDi structural-coherence construct. The graded pattern across external scales—strongest convergence with the most theoretically proximal benchmark—provides initial empirical evidence that the SDi tracks a construct distinguishable from generalized arousal-based distress.

#### 6.8.3 Discriminant and specificity pattern at the facet level (LV1)

To probe whether SDi facets show theoretically expected differential associations, an exploratory series of correlations was computed between each LV1 facet and the DERS subscales and DASS components. Because this analysis comprises 160 tests on *N* = 75–76, results are interpreted as a pattern check rather than as confirmatory evidence; significance is reported with BH correction across the full LV1 grid. A representative subset is reported in Table 16; the full matrix is available from the corresponding author upon reasonable request.

**TABLE 16:** Selected SDi LV1 facet × DERS/DASS subscale correlations illustrating the proximal–distal pattern.

SDi facet (LV1)	External scale/subscale	*N*	*R*	95% CI	*p* (BH)
Fisico (Sé)	DERS clarity	76	–0.77	[–0.85, –0.66]	<0.001
Comportamentale (Q1)	DERS clarity	76	–0.72	[–0.81, –0.59]	<0.001
Disallineamento (Q1)	DERS clarity	76	–0.71	[–0.81, –0.58]	<0.001
Emotivo_ISO (Q1)	DERS clarity	76	–0.71	[–0.81, –0.58]	<0.001
Emotivo_SE (Sé)	DERS clarity	76	–0.70	[–0.80, –0.56]	<0.001
Emotivo_SE (Sé)	DERS awareness	76	–0.55	[–0.69, –0.37]	<0.001
Esistenziale (Q1)	DASS depression	75	–0.48	[–0.63, –0.28]	<0.001
Disallineamento (Q1)	DASS depression	75	–0.69	[–0.79, –0.55]	<0.001
Esistenziale (Q1)	DERS goals	76	–0.06	[–0.28, 0.17]	0.649
Esistenziale (Q1)	DERS impulse	76	–0.06	[–0.28, 0.17]	0.633
Spirituale (Sé)	DERS goals	76	0.02	[–0.21, 0.24]	0.871
Spirituale (Sé)	DERS impulse	76	–0.03	[–0.25, 0.20]	0.807
Energetico (Sé)	DERS impulse	76	0.04	[–0.19, 0.26]	0.768
Mentale (Sé)	DERS impulse	76	0.05	[–0.17, 0.28]	0.665

Upper rows show theoretically proximal pairs (strongest expected); shaded lower rows show theoretically distal pairs (weakest expected). *p*-values are BH-adjusted across the full 160-test exploratory grid.

Two patterns are notable. First, theoretically proximal SDi facets show very strong associations with DERS Clarity in particular (Table 16, upper block). Five SDi facets correlate with DERS Clarity at | *r*| ≥ 0.70: the Fisico facet of Sé organismico (*r* = –0.77), the Comportamentale facet of Isomorfismo (*r* = –0.72), the Disallineamento facet of Isomorfismo (*r* = –0.71), the Emotivo_ISO facet (*r* = –0.71), and the Emotivo_SE facet (*r* = –0.70). This concentration of strong associations on a single DERS subscale is theoretically informative: clarity of emotional experience is the DERS subscale that most closely shares semantic and conceptual ground with the SDi’s “integration as coherence” framing, and it is therefore the subscale where the largest convergent overlap is expected. The SDi facets are not interchangeable with DERS Clarity—they range from *r* = –0.59 to *r* = –0.77 in their associations with it—but the consistency of strong negative correlations across multiple integration facets is consistent with the construct definition.

Second, theoretically distal pairs yield correlations near zero (Table 16, lower block, shaded). The Esistenziale facet of Isomorfismo is uncorrelated with DERS Goals (*r* = –0.06, ns) and DERS Impulse (*r* = –0.06, ns); the Spirituale facet of Sé organismico is uncorrelated with DERS Goals (*r* = 0.02, ns) and DERS Impulse (*r* = –0.03, ns); the Energetico and Mentale facets are uncorrelated with DERS Impulse (*r*s = 0.04 and 0.05, both ns). The graded pattern—strong proximal correlations and near-zero distal ones—supports the interpretation that the SDi facets index differentiated rather than uniform aspects of organismic functioning, and provides discriminant evidence at the facet level that complements the convergent evidence at the LV2 level.

A complementary observation concerns DERS Awareness, which correlated negatively with every SDi facet (rs ranging from –0.42 to –0.61), including the Esistenziale and Spirituale facets. This is consistent with the SDi’s conceptualization of integration as encompassing experiential awareness across multiple domains, and is theoretically distinct from emotion-regulation difficulties as captured by the DERS Goals, Impulse, and Strategies subscales, which showed substantially weaker associations with the same SDi facets. DERS Awareness also correlates only modestly with the other DERS subscales in the present sample (e.g., *r* = 0.16 with DASS Anxiety; *r* = 0.40 with DERS Strategies). The Table 16 illustrates the proximal–distal pattern on selected SDi LV1 facet × DERS/DASS subscale correlations.

#### 6.8.4 Incremental validity: hierarchical regression

To test whether the integration (Q1) and structural-resources (Q2) blocks of the SDi explain variance in wellbeing, distress, and emotion regulation beyond the disharmonies block alone, three sets of hierarchical OLS regressions were estimated (Table 17). For each external outcome, Disarmonie (Q3) was entered at Step 1; Isomorfismo (Q1) at Step 2; and Sé organismico (Q2) at Step 3. For the DERS regression, the Emotivo_SE facet replaced Isomorfismo at Step 2 to test the specificity prediction that emotion-regulation difficulties are most proximally associated with the emotional facet of organismic resources.

**TABLE 17:** Hierarchical regressions predicting external scales from SDi LV2 blocks (*N* = 75 for WHO-5 and DASS Depression; *N* = 75 for DERS).

Outcome/Step	*R* ^2^	Δ*R*^2^	Δ*F* (df)	*p* (ΔF)
WHO-5—Step 1: disarmonie (Q3) only	0.633	–	*F*(1, 73) = 125.71	< 0.001
WHO-5—Step 2: + isomorfismo (Q1)	0.662	0.029	Δ*F*(1, 72) = 6.20	0.015
WHO-5—Step 3: + Sé organismico (Q2)	0.676	0.014	Δ*F*(1, 71) = 3.10	0.083
DASS depression—Step 1: disarmonie (Q3) only	0.561	–	–	–
DASS depression—Step 2: + Isomorfismo (Q1)	0.580	0.019	Δ*F*(1, 72) = 3.25	0.076
DASS depression—Step 3: + Sé organismico (Q2)	0.581	0.001	Δ*F*(1, 71) = 0.16	0.693
DERS total—Step 1: disarmonie (Q3) only	0.625	–	–	–
DERS total—Step 2: + Emotivo_SE (Sé)	0.704	0.078	Δ*F*(1, 72) = 19.05	< 0.001

Δ*R*^2^ values reflect each block’s unique contribution given preceding blocks. For the DERS model, the Emotivo_SE facet replaces Isomorfismo at Step 2 to test the specificity prediction.

For the WHO-5, Step 1 alone explained 63.3% of the variance, Step 2 added 2.9% [Δ*F*(1, 72) = 6.20, *p* = 0.015], and Step 3 added 1.4% as a non-significant trend [Δ*F*(1, 71) = 3.10, *p* = 0.083], yielding a total R^2^ of 0.68. In the final model, Disarmonie remained a significant predictor (*B* = 10.20, *p* = 0.005), Sé organismico approached significance (*B* = 6.61, *p* = 0.083), and Isomorfismo did not reach significance once the other blocks were included (*B* = 6.45, *p* = 0.157). For DASS Depression, Disarmonie alone explained 56.1% of the variance, with Isomorfismo contributing a marginal increment (Δ*R*^2^ = 0.019, *p* = 0.076) and Sé organismico contributing no detectable increment (Δ*R*^2^ = 0.001). For the DERS total, Disarmonie alone explained 62.5% of the variance, and adding the Emotivo_SE facet yielded a substantial and statistically robust increment [Δ*R*^2^ = 0.078, Δ*F*(1, 72) = 19.05, *p* < 0.001], consistent with the theoretical proximity of emotional differentiation to emotion regulation difficulties.

Three qualifications apply. First, the substantial multicollinearity among the SDi LV2 blocks (a feature of the construct rather than a measurement artifact, as documented at the latent level in §6.1) attenuates individual block coefficients in the joint models; the incremental R^2^ values therefore provide a more interpretable index of unique contribution than the individual regression weights. Second, the order of entry was theoretically motivated rather than empirically optimal; reversing the order does not change the total R^2^ but redistributes the incremental variance across steps. Third, the present pattern indicates that Q1 and Q3 contribute robustly to the prediction of wellbeing and emotion regulation, while Q2 contributes mainly through Q1—a finding that is theoretically coherent with the structural model in §6.3, in which Q2 is modeled as upstream of Q1 in the sequence Q2 → Q1 → Q3. The trend-level Q2 increment is therefore consistent with, rather than at odds with, the structural architecture reported earlier. Hierarchical regressions predicting external scales from SDi LV2 blocks are illustrated in the Table 17.

#### 6.8.5 Summary of convergent evidence

Three classes of evidence emerge from the convergent validity analyses. First, the four SDi LV2 composites correlate substantially with the WHO-5 in the predicted positive direction and with the DASS-21 and DERS in the predicted negative direction, with all 24 primary correlations surviving BH correction and the strongest associations falling on the most theoretically proximal benchmarks. Second, theoretically proximal SDi facet × external subscale pairs yield strong correlations (notably with DERS Clarity, where five facets correlate at | *r*| ≥ 0.70), while theoretically distal pairs yield correlations near zero, supporting the differentiation of the LV1 facets. Third, Disarmonie and Isomorfismo contribute robustly to the prediction of wellbeing and depression beyond any single block alone, and the emotional-resources facet contributes substantially (Δ*R*^2^ = 0.078, *p* < 0.001) to the prediction of emotion regulation difficulties beyond Disarmonie. Interpretive implications are discussed in § 7.7.

## 7 Discussion

The present study sought to operationalize psychological integration as a multi-level organismic architecture and to empirically test its structural coherence using the Self-Development Inquiry (SDi). Across measurement, mediation, and network analyses, the findings provide convergent evidence that psychological functioning may be meaningfully understood as a structured organization linking embodied resources, integrative processes, and dysregulation signals. Importantly, the results do not merely support the statistical adequacy of the model; they illuminate specific psychological processes that appear central to adaptive functioning and distress regulation.

### 7.1 Model fit and convergent validity considerations

Under ordinal-appropriate estimation (WLSMV with polychoric correlations), the incremental fit indices for the first-order correlated measurement model (CFI = 0.943; TLI = 0.933) approach the 0.95 benchmark for excellent fit and exceed conventional thresholds for adequate fit. RMSEA (0.065) and SRMR (0.053) indicate good approximate and residual fit. The trimmed structural model retained acceptable fit (CFI = 0.924, TLI = 0.915, RMSEA = 0.075, SRMR = 0.065). These indices represent a substantial improvement over a maximum-likelihood specification on the same data, consistent with methodological recommendations that ordinal Likert items be modeled with polychoric correlations rather than treated as continuous (Li, 2016; Wu and Estabrook, 2016). Even allowing that the structural model includes a sizeable number of latent constructs distributed across three theoretically distinct levels, a configuration in which CFI/TLI are known to be comparatively conservative (Kenny and McCoach, 2003), the obtained indices support the adequacy of the SDi measurement architecture. CFI/TLI values in the 0.92–0.94 range are commonly reported for multidimensional ordinal-data instruments of comparable structural complexity under WLSMV estimation (Marsh et al., 2004), and the SDi meets adequate-fit thresholds while approaching excellent-fit benchmarks under estimation conditions appropriate for ordinal data. Comparisons against unidimensional, second-order, and bifactor alternatives further confirm the first-order correlated structure as the most defensible specification.

Convergent validity is supported by composite reliability values that are acceptable to strong across constructs (ω = 0.68 to 0.92, with most ω ≥ 0.77) and Average Variance Extracted (AVE) values that are satisfactory for the majority of constructs. The single residual concern is ISO_CEX (AVE = 0.48), which approaches but does not reach the 0.50 benchmark. Methodological work has noted that AVE values in the 0.40–0.50 range remain acceptable when composite reliability exceeds 0.60 (Fornell and Larcker, 1981; Lam, 2012); ISO_CEX meets this condition (ω = 0.73). In our reading, this pattern reflects the construct bandwidth of the SDi domains: unlike narrow trait measures designed to maximize item homogeneity, the SDi targets organismic and integrative processes that are broad and multi-faceted. Inter-item heterogeneity is therefore expected, given that “integration” is theorized as coordination across differentiated experiential channels rather than as a unidimensional tendency expressed through redundant items. Discriminant validity is preserved under a conservative HTMT criterion (all values < 0.85), confirming that the constructs are empirically distinguishable. Taken together (strong composite reliability, satisfactory AVE for the majority of constructs, preserved discriminant validity, and adequate-to-good model fit) the SDi provides a defensible psychometric operationalization of a complex organismic–transpersonal architecture (Kline, 2023). Multicollinearity diagnostics (composite- and latent-level VIFs all below 3) further confirm that the substantial Q2–Q1 association does not compromise the stability of structural estimates.

### 7.2 Psychological integration as structured coherence

The theoretical framework advanced earlier conceptualized integration not as elevated mood or subjective satisfaction, but as coherence across differentiated experiential domains: affective, cognitive, somatic, relational, and existential. This view is consistent with organismic psychology, embodied cognition, and phenomenological psychiatry, all of which converge on the notion that wellbeing reflects coordination rather than symptom absence.

The structural results support this conceptualization.

Organismic resources were not globally associated with outcomes; instead, each resource showed differentiated associations with specific integration processes. Physical–cognitive organization (SE_FIS) was strongly associated with both mental integration and external coherence. Emotional organization (SE_EMO) was primarily associated with mental integration; spiritual organization (SE_SPI) was associated with internal coherence. These differentiated patterns are consistent with developmental and humanistic models that conceptualize distinct structural capacities as underpinning distinct modes of integration.

Psychologically, this pattern is consistent with the readings that:

- Cognitive clarity and embodied grounding correspond with behavioral congruence.
- Emotional differentiation corresponds with reflective regulation.
- Spiritual orientation corresponds with internal existential alignment.

Such differentiation aligns with the literature’s emphasis on “differentiation without fragmentation” (Siegel, 2001) and challenges reductionist approaches that collapse wellbeing into single-factor indices.

### 7.3 External coherence and psychological outcomes

Among all integration domains, external coherence (ISO_CEX) defined as alignment between thought, feeling, and action, was the strongest proximal correlate of both physical and mental disharmonies.

This pattern has direct psychological implications.

Physical disharmonies in the SDi include fatigue, somatic tension, disconnection from bodily signals, and neglect of basic needs. Mental disharmonies include repetitive negative thinking, confusion, loss of meaning, and existential disorientation.

The strong negative cross-sectional associations between external coherence and both forms of dysregulation are consistent with a reading of enacted congruence as a central regulatory correlate. Participants whose reported behavior was aligned with their reported internal states also reported less somatic strain and less cognitive–existential fragmentation.

This pattern resonates with Rogers’ concept of congruence (Thorne, 2003), Gendlin’s emphasis on embodied alignment (1991), and phenomenological psychiatry’s account of structural disorganization (Fuchs, 2020). The associational evidence is consistent with the theoretical reading that experiential alignment is psychologically protective; whether such alignment causally buffers dysregulation remains an empirical question requiring longitudinal data.

Thus, psychological integration appears within the present cross-sectional results not merely as an internal reflective capacity but as a multi-component construct that includes behavioral alignment.

### 7.4 Mediation pathways and the role of embodied organization

Bootstrapped mediation analyses further characterized this structure. Physical–cognitive organization (SE_FIS) showed large statistical indirect effects on both physical (DIS_FIS) and mental disharmonies (DIS_MEN) via external coherence (ISO_CEX). In practical terms, individuals reporting greater embodied stability also reported greater behavioral congruence and, in turn, lower dysregulation; the mediation evidence is statistical, not causal.

This pattern is conceptually coherent with embodied cognition models and polyvagal-informed regulation theories, which emphasize the role of physiological grounding in adaptive action (Porges, 2025). The cross-sectional evidence is consistent with the theoretical reading that bodily stability and cognitive organization correspond with the alignment of internal experience and behavior, although establishing this as a causal sequence requires longitudinal data.

The mediation was partial: energetic organization (SE_ENE) retained a direct association with physical disharmony that was not accounted for by the integration mediators. This pattern is consistent with embodied regulation perspectives in which vitality-related processes (e.g., fatigue, activation, physiological energy) are associated with dysregulation through both integrative and direct channels, and indicates that integration is not the sole proximal correlate of dysregulation.

The position of emotional organization (SE_EMO) within the structural model warrants closer reading. The direct path to physical disharmony was positive (β = +0.21), while the indirect path via mental integration was negative (β = -0.07), yielding a small positive total effect (β = +0.14). The opposite signs of these two components identify an inconsistent-mediation or suppression pattern, which is theoretically substantive rather than statistically anomalous. Two distinct mechanisms appear to operate simultaneously: emotional differentiation supports reflective regulation, which is the protective channel transmitted through ISO_MEN; at the same time, heightened emotional awareness plausibly increases interoceptive sensitivity to bodily signals, amplifying the perception of somatic strain. These dual mechanisms are consistent with constructionist accounts of emotion, which characterize emotional granularity as a regulatory resource that simultaneously enhances awareness of internal bodily states (Barrett, 2017; Khalsa et al., 2018). Emotional openness, in this reading, is not uniformly protective: its adaptive value depends on whether the heightened sensitivity it confers is accompanied by sufficient integrative capacity to translate awareness into regulation.

### 7.5 Disharmonies as distributed regulatory signals

The complementary network analysis adds depth to the structural findings. Disharmony indicators formed a densely interconnected network with high clustering and a stable edge structure. This suggests that dysregulation signals interact conditionally rather than functioning solely as reflective outputs of a single latent disorder (hypothesis for longitudinal testing).

Psychologically, this network architecture is consistent with the possibility that fatigue, cognitive confusion, loss of meaning, and somatic tension are mutually reinforcing in the experiential domain, although causal direction cannot be inferred from cross-sectional data. The bridge structure, with persistent unexplained fatigue and cognitive disconnection occupying the strongest bridge positions, is consistent with phenomenological accounts in which disturbances in embodied experience are linked to disturbances in meaning and identity, though the temporal sequence of these links remains an open empirical question.

The descriptive convergence between SEM and network findings is theoretically suggestive. The hierarchical model captures the cross-sectional associations among structural resources, integration, and dysregulation; the network model captures the conditional interdependence among dysregulation indicators themselves. Together, they are consistent with a depiction of psychological integration as a dynamic, multi-level system rather than a static trait, a depiction that requires longitudinal data for proper empirical evaluation.

### 7.6 Magnitude of explained variance

The substantial proportion of variance explained in the disharmony outcomes (DIS_FIS *R*^2^ = 0.71; DIS_MEN *R*^2^ = 0.72) merits comment. Within the SDi architecture, dysregulation is theorized as the integrated downstream signal of upstream structural and integrative processes; substantial explanatory coverage is therefore conceptually expected rather than indicative of overfitting. Three lines of evidence rule out the alternative interpretations.

First, multicollinearity is not the source: variance inflation factors at both the composite and latent levels remained below 3, with a condition number of 9.84—values that are well below the thresholds at which redundancy could artificially inflate explained variance. Second, overfitting is not the source: the cross-sample CFA fit indices reported above were virtually identical between the confirmatory half and the full sample, indicating that the structural relations generalize beyond the derivation subsample. Third, the explanatory pattern is graded: variance explained is highest at the regulatory endpoint (Q3), intermediate at the integration mediators (Q1), and lowest at internal coherence (ISO_CIN *R*^2^ = 0.46), consistent with a hierarchical architecture in which dysregulation aggregates upstream contributions while integration mediators retain genuine variance not captured by their structural antecedents.

The high explanatory power therefore reflects the theoretically expected aggregation of multi-level inputs at the regulatory endpoint, supported empirically by VIF, cross-sample stability, and a graded R^2^ pattern across levels of the hierarchy. External replication on independent samples remains the appropriate next step, as discussed in the Limitations.

### 7.7 Convergent and discriminant validity considerations

The convergent evidence reported in §6.8 supports the interpretive claim advanced throughout this paper: that the SDi indexes a structurally organized construct whose external correlates are theoretically coherent rather than diffuse. Three observations bear directly on the construct-validity status of the instrument.

First, the magnitude and direction of the LV2 correlations align with the architecture proposed in §4 and instantiated in the structural model of §6.3. Disarmonie correlates positively with all three distress measures and negatively with the WHO-5; Isomorfismo and Sé organismico correlate in the opposite direction across the same external scales. The strongest external correlations fall on the WHO-5 and DASS Depression—the two instruments most closely aligned with the SDi’s framing of integration as organismic coherence and disharmonies as demoralization-related signals—while the weakest associations fall on DASS Anxiety, which captures autonomic-arousal phenomena less directly relevant to the structural-coherence construct. The graded ordering across external scales would not be expected if the SDi simply indexed generalized affective distress; the differential strength across benchmarks supports a more specific construct interpretation.

Second, the facet-level analyses provide a discriminant-validity pattern that the LV2 analyses cannot. At the facet level, the SDi’s integration components correlate strongly with DERS Clarity (five facets at | *r*| ≥ 0.70), consistent with the conceptual proximity between emotional clarity and the SDi’s coherence framing, but they correlate near zero with DERS Goals and DERS Impulse for the most theoretically distal facets (Esistenziale, Spirituale, Energetico, Mentale). This selectivity argues against a unidimensional account of the SDi: if the instrument were measuring a single global construct, all facets would correlate similarly with all DERS subscales. The observed dissociation between strong proximal and near-zero distal correlations is consistent with the multidimensional architecture proposed in the theoretical framework.

Third, the hierarchical regression results characterize the relations among the SDi LV2 blocks themselves. Disarmonie consistently emerges as the dominant predictor of external outcomes, with Isomorfismo contributing modest but detectable additional variance and Sé organismico contributing only at trend level. This pattern is theoretically coherent rather than anomalous: the structural model places Sé organismico upstream of Isomorfismo, with Sé organismico’s contribution to dysregulation-related outcomes flowing primarily through the integrative pathways of Q1. When Q1 is already in the regression, Q2’s incremental contribution is therefore expected to be attenuated—and the empirical pattern matches this expectation. The robust incremental contribution of the Emotivo_SE facet to DERS prediction (Δ*R*^2^ = 0.078) further indicates that emotional differentiation, conceptualized as a structural resource, captures variance in emotion-regulation difficulties not accounted for by Disarmonie alone, supporting the theoretical specificity of the emotional facet within the Q2 block.

Two interpretive cautions accompany these findings. The substantial WHO-5 correlation (*r* = 0.82) is consistent with the SDi’s conceptualization as an organismic-coherence index but invites the reasonable question of whether the instrument is empirically distinguishable from a comprehensive wellbeing measure. Several lines of evidence in the present manuscript argue that it is. The structural model of §6.3 demonstrates internal architecture (resources → integration → disharmonies) that no single-factor wellbeing instrument captures; the network analysis of §6.6 documents conditional interdependencies among disharmony indicators inconsistent with reflective unidimensional measurement; and the facet-level discriminant pattern in §6.8 shows that SDi facets correlate selectively with DERS subscales rather than uniformly. Direct comparison against multidimensional eudaimonic instruments (e.g., the Ryff Scales of Psychological Wellbeing; Ryff, 1989) would provide a stronger discriminant test and is identified as a priority for future validation work.

The cross-sectional design also constrains interpretation. The convergent associations reported here describe co-occurring patterns at a single time point and do not establish whether changes in SDi scores precede, follow, or are independent of changes in wellbeing, distress, or emotion regulation. Whether the SDi is sensitive to within-person change over time, and whether changes in integration domains predict subsequent reductions in dysregulation indicators, are empirical questions that require longitudinal designs and are flagged in §7.10 as priority directions for future research. The present analyses establish that the SDi behaves as predicted in cross-section against well-validated benchmark instruments; longitudinal validation of the temporal architecture remains a separate empirical step.

Taken together, the convergent and discriminant evidence reported in §6.8 strengthens the case that the SDi operationalizes a multidimensional, structurally organized construct with theoretically coherent external correlates. The instrument shows convergent validity with established wellbeing, distress, and emotion-regulation benchmarks; differentiated validity at the facet level; and incremental contributions consistent with the upstream–downstream logic of the structural model. These properties, combined with the internal psychometric structure documented in §6.1–§6.7, position the SDi as a defensible operationalization of psychological integration within the organismic–transpersonal framework, while leaving longitudinal validation and direct comparison against eudaimonic wellbeing measures as appropriate next steps.

### 7.8 Clinical usefulness and applied implications

Although the present study is cross-sectional and psychometric in nature, several clinically relevant implications emerge.

#### 7.8.1 Assessment of multi-level functioning

The SDi provides a structured means of assessing:

- Organismic capacities (structural resources),
- Integration processes (functional coherence),
- Dysregulation signals (experiential fragmentation).

Rather than measuring only symptom severity, clinicians may use the SDi to identify whether distress is primarily associated with:

- Deficits in structural resources (e.g., low vitality),
- Breakdown in integration (e.g., behavioral incongruence),
- Self-reinforcing dysregulation loops.

This may support formulation-based interventions rather than purely symptom-focused approaches.

### 7.8.2 Targeting behavioral congruence

Given the central role of external coherence, interventions that enhance alignment between internal states and behavior may exert broad regulatory effects. Such interventions may include:

- Experiential and emotion-focused therapies,
- Body-oriented psychotherapies,
- Acceptance-based approaches targeting value-congruent action,
- Psychosynthesis or transpersonal integration work.

The findings suggest that behavioral alignment may function as a leverage point capable of influencing both somatic and cognitive–existential distress.

#### 7.8.3 Differentiating regulatory pathways

The partial mediation findings imply that not all dysregulation flows through integration processes. For example:

- Energetic deficits may require direct somatic or lifestyle interventions.
- Emotional differentiation may need containment and integrative scaffolding.

Thus, the SDi may assist clinicians in distinguishing when to focus on embodied regulation, cognitive integration, existential orientation, or behavioral congruence.

#### 7.8.4 Monitoring change

The relatively high explained variance in disharmonies suggests that changes in integration domains may meaningfully relate to reductions in distress. While longitudinal validation is required, the SDi may eventually serve as an outcome monitoring tool in integrative or organismic-based therapeutic settings.

### 7.9 Theoretical contributions

The findings advance the literature reviewed earlier by empirically characterizing integration through three structural patterns:

- Cross-sectional associations consistent with a hierarchical organization across levels (resources, integration, and dysregulation).
- Partial rather than full statistical mediation of the resource–dysregulation relationship by integration processes.
- Conditional interdependence among dysregulation indicators, examined via network psychometrics.

This multi-model approach bridges organismic psychology, phenomenological psychiatry, complexity theory, and contemporary psychometrics, and contributes to the operationalization of integration as a measurable structural construct rather than a metaphor.

The data are also consistent with the conceptualization that integration is not synonymous with subjective happiness; rather, it reflects structured coordination across experiential systems. Within the present cross-sectional results, fatigue, cognitive confusion, loss of meaning, and somatic disconnection are associated with reduced enacted coherence. The conceptual distinction between integration and wellbeing is supported empirically by the convergent and discriminant pattern reported in §6.8 and §7.7, in which the SDi tracks wellbeing and distress benchmarks substantively but exhibits a graded and differentiated pattern of associations inconsistent with reduction to any single external construct.

### 7.10 Measurement equivalence across gender

The SDi demonstrated full configural and metric invariance and partial scalar invariance across gender. Under the primary ordinal estimator (WLSMV with polychoric correlations), fit-index changes for both metric and scalar models fell well within the Chen (2007) thresholds for invariance, indicating that loadings, thresholds, and intercepts are practically equivalent across women and men. Under the supplementary continuous estimator (MLR), the χ^2^ difference test rejected the equality of intercepts, but this divergence is consistent with the well-documented sample-size sensitivity of the χ^2^ test in large samples (Cheung and Rensvold, 2002): the underlying intercept differences are small in absolute terms, as confirmed by the negligible fit-index changes. Item-level diagnostics identified a small number of items with modest intercept differences (Putnick and Bornstein, 2016).

Two implications follow. First, the structural relations among organismic resources, integration processes, and dysregulation signals (the central architecture of the SDi) operate equivalently in women and men. Second, latent-mean comparisons across gender, while not the focus of the present manuscript, would be warranted under a partial scalar specification in which the items identified above are freed (Byrne et al., 1989). The earlier conclusion that scalar invariance was not achieved (a) was based on a continuous-data estimator that is not optimal for ordinal items, and (b) understated the practical equivalence of the measurement model: under the appropriate ordinal estimator and contemporary fit-index criteria, the SDi shows considerably more cross-gender stability than the original analysis suggested.

### 7.11 Limitations and future directions

Several limitations constrain interpretation of the present findings.

First, the cross-sectional design precludes causal inference. Although the structural model is statistically consistent with a hierarchical mediation sequence, temporal ordering cannot be established. It remains possible that dysregulation influences integration processes, or that bidirectional effects operate dynamically over time. Although a 50/50 random split between exploratory and confirmatory subsamples reduces the risk of sample-specific overfitting, full external replication on an independent sample remains a necessary next step.

Second, reliance on self-report methodology introduces potential common-method variance and subjective bias. Constructs such as integration and dysregulation are inherently experiential, yet future multimethod approaches (e.g., behavioral tasks, physiological indices, clinician ratings) would strengthen construct validity. Third, the sample was non-clinical and demographically imbalanced, limiting generalizability to populations experiencing clinically significant distress. The high explained variance in disharmonies, while theoretically coherent, may differ in clinical samples characterized by greater heterogeneity and symptom severity.

Third, mediation pathways were not tested longitudinally; thus, the proposed organismic sequence (resources → integration → dysregulation) should be regarded as a theoretically informed structural hypothesis rather than a confirmed developmental process.

Fourth, while the convergent validity analyses reported in §6.8 establish that the SDi correlates with established wellbeing, distress, and emotion-regulation benchmarks in theoretically expected directions, this evidence is based on a sub-sample (*N* = 76) drawn from the same population frame as the main sample. Full external replication on independent and demographically distinct samples remains a necessary next step. Direct discriminant comparison against multidimensional eudaimonic wellbeing measures (e.g., the Ryff Scales of Psychological Wellbeing; Ryff, 1989) is identified as a priority for further validation work.

Future research should additionally prioritize longitudinal and prospective designs capable of modeling temporal dynamics among organismic resources, integration processes, and dysregulation signals. Experience-sampling or repeated-measures designs would allow examination of whether increases in integration are followed by reductions in disharmonies and whether dysregulation activation feeds back into structural capacities. Clinical studies are needed to assess sensitivity to psychotherapeutic change and to determine whether integration domains operate as mechanisms of treatment response. Cross-cultural validation and measurement invariance testing across sociocultural contexts will be essential to establish the generalizability of the SDi.

Additionally, extending dynamic network modeling to longitudinal data is necessary to clarify whether and how dysregulation loops evolve over time and whether specific integration domains dampen network activation or disrupt bridge pathways between somatic and cognitive–existential distress. The cross-sectional networks reported here describe regularized conditional associations at a single time point and do not establish causal direction, generative mechanism, or temporal stability of bridge structures; longitudinal designs are required to address these questions. Together, such research would move the SDi framework from cross-sectional structural coherence toward a temporally validated model of organismic regulation.

## 8 Conclusion

This study operationalized psychological integration as a multi-level organismic architecture linking structural resources, functional coherence, and dysregulation signals. The findings provide convergent support for this framework across measurement, mediation, network, and convergent validity analyses.

First, the SDi demonstrated acceptable psychometric adequacy within a theoretically ambitious multidimensional structure. Despite conservative incremental fit indices, approximate fit, reliability, discriminant validity, and theoretically coherent loadings collectively support the measurement foundation.

Second, the structural findings characterize the cross-sectional architecture of integration. Organismic resources did not show diffuse global associations with outcomes; instead, they showed differentiated associations with specific integration domains. Physical–cognitive organization emerged as a central structural correlate, primarily through its strong association with external coherence (alignment between thought, feeling, and action). External coherence, in turn, was the dominant proximal correlate of both somatic and cognitive–existential disharmonies. This pattern is consistent with long-standing organismic and congruence-based theories that position enacted alignment as central to psychological health.

Third, mediation analyses supported a partial hierarchical pattern. Structural capacities were associated with dysregulation largely via the integrative mediators, while energetic vitality retained direct associations not accounted for by integration, underscoring the embodied and non-reductive nature of psychological organization. These findings are consistent with the conceptualization of integration as neither purely cognitive nor purely affective but as coordinated system-level functioning.

Fourth, network analysis showed that dysregulation indicators form a densely interconnected and stable system of conditional associations. The identification of bridge nodes linking somatic and existential indicators is consistent with phenomenological accounts of structural disorganization. Whether dysregulation propagates through feedback loops once activated, and whether integration processes function as system-wide dampening mechanisms, are hypotheses requiring longitudinal data for evaluation.

Fifth, convergent validity analyses against the WHO-5, DASS-21, and DERS demonstrated that the SDi correlates with established wellbeing, distress, and emotion-regulation benchmarks in theoretically expected directions and magnitudes. The graded pattern of associations showing strongest convergence with the most theoretically proximal benchmarks (wellbeing, depression), weakest with anxiety, supports the interpretation that the SDi indexes structural coherence rather than generalized affective distress. Facet-level analyses provide complementary discriminant evidence, with theoretically proximal SDi facet × subscale pairs yielding strong correlations and theoretically distal pairs yielding correlations near zero, supporting the multidimensional differentiation of the instrument.

Taken together, the results position psychological integration as:

- Hierarchically organized across structural, functional, and regulatory levels,
- Partially mediated rather than strictly sequential,
- Dynamically sustained through network interdependence at the dysregulation level.
- Empirically convergent with established wellbeing, distress, and emotion-regulation benchmarks while remaining differentiated at the facet level.

Importantly, the SDi moves integration beyond metaphor. Rather than equating wellbeing with positive affect or life satisfaction, the present findings conceptualize psychological health as structured coherence across embodied, cognitive, relational, and existential domains. The convergent evidence reported here supports this conceptualization empirically: the SDi tracks wellbeing, distress, and emotion-regulation benchmarks substantively while exhibiting a graded and differentiated pattern of associations inconsistent with reduction to any single external construct.

Future longitudinal and clinical research will be required to establish temporal directionality and sensitivity to therapeutic change. The current cross-sectional evidence, combining internal psychometric structure, structural and network modeling, and convergent validity against established external benchmarks, positions the SDi as a theoretically grounded and empirically coherent instrument capable of advancing organismic–transpersonal models of psychological functioning within contemporary psychometric standards.

## Data Availability

The raw data supporting the conclusions of this article will be made available by the authors, without undue reservation.
